# Two Novel Bacterial Species, *Rhodanobacter lycopersici* sp. nov. and *Rhodanobacter geophilus* sp. nov., Isolated from the Rhizosphere of *Solanum lycopersicum* with Plant Growth-Promoting Traits

**DOI:** 10.3390/microorganisms12112227

**Published:** 2024-11-03

**Authors:** Haejin Woo, Inhyup Kim, Geeta Chhetri, Sunho Park, Hyunji Lee, Subin Yook, Taegun Seo

**Affiliations:** Department of Life Science, Dongguk University, Goyang 10326, Republic of Korea; woohj999@dongguk.edu (H.W.); duckling91@dgu.ac.kr (I.K.); lucky_salman@dongguk.edu (G.C.); eksvnd97@dgu.ac.kr (S.P.); guswl4851@dgu.ac.kr (H.L.); qw745043@dgu.ac.kr (S.Y.)

**Keywords:** *Rhodanobacter*, plant growth-promoting bacteria, pH tolerance, genomic analysis, rhizosphere, novel species, polyphasic taxonomy

## Abstract

Two novel bacterial species were isolated from the rhizosphere of *Solanum lycopersicum* (tomato plant), both exhibiting plant growth-promoting properties. Two isolated strains, *Rhodanobacter lycopersici* sp. nov. Si-c^T^ and *Rhodanobacter geophilus* sp. nov. S2-g^T^, were classified through a polyphasic approach, confirming their novel status within the *Rhodanobacter* genus. The strains demonstrated a remarkable tolerance to extreme pH conditions, with *R*. *lycopersici* Si-c^T^ surviving in pH 3.0–13.0 and *R*. *geophilus* S2-g^T^ tolerating pH 2.0–13.0. Additionally, both strains exhibited multiple plant growth-promoting traits, including indole-3-acetic acid and ammonia production, phosphate solubilization, and siderophore formation. These characteristics suggest that the two strains may play an important role in promoting plant growth, especially in soils with variable pH levels. However, since the direct impact on plant growth was not experimentally tested, the potential of these bacteria for agricultural applications remains to be confirmed through further research. This study expands our understanding of the diversity within the *Rhodanobacter* genus and provides insights into the potential use of these novel species in sustainable agriculture.

## 1. Introduction

In response to the increasing global population and rising demand for crops, there is growing attention on sustainable farming practices [1]. The overuse of chemical fertilizers has caused significant soil degradation and environmental pollution, raising concerns about ecosystem health and food security [2]. As a result, plant growth-promoting bacteria (PGPB) have emerged as a promising solution, offering eco-friendly alternatives to enhance plant growth without the harmful effects of chemical inputs [3,4]. Several genera, including *Acinetobacter*, *Agrobacterium*, *Arthrobacter*, *Bacillus*, *Burkholderia*, *Enterobacter*, *Flavobacterium*, *Lysinibacillus*, *Paenibacillus*, and *Pseudomonas*, have been reported to contain PGPB that positively influence plant health and growth [5]. Certain PGPB are known to thrive under harsh conditions, such as salt, temperature, and drought stress [6,7,8]. PGPB act directly as biofertilizers by stimulating root growth, restoring root nodules, controlling plant stress, and breaking down heavy metals in the soil while also contributing indirectly through antimicrobial activity, induction of systemic resistance, and nutrient competition [9,10].

Plant growth-promoting rhizobacteria (PGPR), a subset of PGPB, are soil bacteria that specifically colonize the plant root surface and rhizosphere, contributing to plant growth [3,11]. These bacteria actively interact with the plants in the root environment, promoting growth through mechanisms such as enhancing nutrient uptake, suppressing pathogens, and producing hormones [12]. Rhizobacteria in the rhizosphere can colonize both the root surface and surrounding areas, influencing plant growth under suitable soil and environmental conditions [5,13,14,15].

In this context, this study focused on isolating and identifying efficient strains of rhizobacteria from the tomato rhizosphere, aiming to secure natural microbial resources. Two novel *Rhodanobacter* species were isolated, and their phylogenetic, physiological, and chemotaxonomic attributes were assessed. These strains were evaluated for key plant growth-promoting traits, such as indole-3-acetic acid (IAA) production, ammonia production, siderophore production, nitrogen fixation, and phosphate solubilization. Although these properties were evaluated in vitro, further research is required to assess their effectiveness in vivo. Despite the need for additional studies, this research marks an important step toward uncovering novel microbial bioresources and expanding our understanding of *Rhodanobacter* species. It lays the groundwork for developing strains that could contribute to sustainable and eco-friendly agricultural practices.

## 2. Materials and Methods

### 2.1. Isolation and Culture Conditions

Strains Si-c^T^ and S2-g^T^ were isolated from the rhizosphere of potted tomato plants at Dongguk University, Ilsan, Republic of Korea (37°40′42.8″ N, 126°48′24.9″ E). Before planting in a pot, tomato seeds were surface sterilized following the previously described method [16]. Five seeds were planted in a round pot (diameter, 18.5 cm; height, 5 cm; base, 13 cm) with 400 g of Plant World-Mix soil substrate (Nongwoo Bio Co., Ltd., Suwon, Republic of Korea). The soil contained 49.876% coco peat, 25% peat moss, 12% perlite, 7% vermiculite, 6% zeolite, and 0.11% NPK fertilizer containing 0.187 g/kg of nitrogen, phosphorus, and potassium. The soil pH is 5.5–6.0. One week after planting, all seedlings were removed, leaving the healthiest seedling. The remaining seedling was grown with regular irrigation using tap water for six months.

For screening bacterial colonies, the soil samples were prepared using previously described methods [17,18]. The tomato plant was uprooted from the pot, and the soil on the roots was vigorously shaken off and washed with running tap water to remove any non-adhering soil. A total of 2 g of soil surrounding the roots was transferred into a 50 mL conical tube along with 5 mL of sterilized 0.85% (*w*/*v*) NaCl. Subsequently, the rotated mixture was diluted to 10^−1^–10^−4^ using the same reagent, and 100 μL of each dilution was spread on Reasoner’s 2A (R2A; MB Cell, Seoul, Republic of Korea) agar that was incubated at 30 °C for 3 days. Strains Si-c^T^ and S2-g^T^ were repeatedly re-streaked on the same medium to ensure purity. Pure colonies of each strain were stored at −80 °C in a 25% (*v*/*v*) glycerol mixture, prepared by combining R2A broth and 50% glycerol in a 1:1 ratio for long-term preservation. Strains Si-c^T^ and S2-g^T^ have been deposited in culture collections under the accession numbers KACC 23732^T^ and TBRC 19125^T^ for Si-c^T^ and KACC 23733^T^ and TBRC 19012^T^ for S2-g^T^.

### 2.2. Genomic Analysis and Genome Annotation

The genomic DNA of strains Si-c^T^ and S2-g^T^ was extracted using the Maxwell^®^ RSC Tissue DNA Kit (Promega, Madison, WI, USA). The samples were then prepared for sequencing with the TruSeq Nano DNA Library Prep Kit (Illumina, Inc., San Diego, CA, USA) and sequenced on the Illumina NovaSeq 6000 platform. The sequencing data were assembled using the SPAdes ver. 3.15.0. de novo assembler [19] at Macrogen (Seoul, Republic of Korea). The completeness and contamination rates of the genomes were identified using the CheckM bioinformatics tool (https://ecogenomics.github.io/CheckM, accessed on 10 September 2024) [20]. The phylogenomic trees were reconstructed using the 92 up-to-date bacterial core gene sets (UBCG) and EzAAI [21,22]. The draft genomes were annotated using both the NCBI Prokaryotic Genome Automatic Annotation Pipeline (PGAP) and the Rapid Annotation using Subsystem Technology (RAST) online server [23,24]. Genes related to motility, pH tolerance, and plant growth-promoting traits were identified through the PGAP platform. Prokka [25] was used to annotate the genome and identify coding sequences (CDSs) and features, such as mobile genetic elements, comprehensive antibiotic resistance genes, CRISPRs, and phages [26,27,28]; these were visualized using a circular genomic map created with the Proksee online tool (https://proksee.ca/, accessed on 31 July 2024) [29]. Orthologous clusters (OCs) were compared between the isolated strains and closely related species using the OrthoVenn 3.0 online platform [30], with protein sequences from the annotated genomes processed using PGAP. The antibiotics and Secondary Metabolite Analysis Shell (antiSMASH) ver. 7.1.0. was employed to identify biosynthetic gene clusters (BGCs) associated with secondary metabolite compounds in the novel strains using the relaxed setting [31]. The abbreviations used for interpreting the antiSMASH results are defined in the antiSMASH glossary (https://docs.antismash.secondarymetabolites.org/glossary/, accessed on 11 September 2024). The overall genome relatedness index (OGRI) between the isolated strains and closely related *Rhodanobacter* species was calculated by using EzAAI for average amino acid identity (AAI), OrthoANI (www.ezbiocloud.net/tools/orthoani, accessed on 24 June 2024) for average nucleotide identity (ANI), and the Genome-to-Genome Distance Calculator ver. 3.0 (https://ddgc.dsmz.de/, accessed on 24 June 2024) for digital DNA–DNA hybridization (dDDH) [22,32,33]. The Whole Genome Shotgun projects for strains Si-c^T^ and S2-g^T^ have been deposited at DDBJ/ENA/GenBank under the accession numbers JBFOHK000000000 and JBFOHL000000000, respectively.

### 2.3. 16S rRNA Gene Sequencing and Phylogenetic Analysis

For phylogenetic analysis, this study sequenced the 16S rRNA genes of Si-c^T^ and S2-g^T^ using the Sanger method and retrieved the full-length 16S rRNA sequences from the genomes of these strains. The genome-derived 16S rRNA sequences were used for all phylogenetic analyses. The 16S rRNA genes of strains Si-c^T^ and S2-g^T^ were amplified using universal primer set 27F (5′-AGA GTT TGA TCC TGG CTC AG-3′)/1492R (5′-GGT TAC CTT GTT ACG ACT T-3′) [34] and 518F (5′-CCA GCA GCC GCG GTA ATA C-3′)/805R (5′-GAC TAC CAG GGT ATC TAA TC-78 3′) [35] and sequenced by Sanger method at Solgent Co., Ltd. (Daejeon, Republic of Korea). PCR was performed on an ABI PRISM 3730XL DNA Analyzer (Applied Biosystems, Waltham, MA, USA) with 30 cycles of denaturation at 96 °C for 10 s, annealing at 50 °C for 5 s, and extension at 60 °C for 4 min. The reaction mixture contained 4 µL of Terminator Ready Reaction Mix, 1 µL of cleaned PCR product (20–40 ng), 1 µL each of forward and reverse primers (5 pmol), and sterile water to a final volume of 10 µL. The amplicons were compiled using SeqMan software version 5.0 (DNASTAR, Madison, WI, USA). Nearly full-length 16S rRNA gene sequences of the two strains were obtained using the Sanger method, and full-length 16S rRNA gene sequences were retrieved from the draft genomes using ContEst16S [36]. The sequences from both methods were compared using the NCBI nucleotide BLAST server [37]. The 16S rRNA gene sequences obtained from the genome were uploaded to the EzBioCloud server (www.ezbiocloud.net/, accessed on 10 September 2024) [38] to identify phylogenetic neighbors based on 16S rRNA gene sequences of type species with valid names. Phylogenetic analysis was performed using phylogenetic trees constructed with MEGA version X software [39] by employing the maximum likelihood (ML), neighbor-joining (NJ), and maximum parsimony (MP) algorithms [40,41,42]. The confidence level of tree topologies was determined through bootstrap analysis with 1000 replicates [43]. *Xanthomonas campestris* ATCC 33913^T^ was used as an outgroup. The GenBank accession numbers for the 16S rRNA gene sequences of strain Si-c^T^ are PP647361 (Sanger method) and PP946760 (whole genome). For strain S2-g^T^, the accession numbers are PP647365 (Sanger method) and PP946762 (whole genome).

### 2.4. Physiological and Morphological Analysis

Physiological and chemotaxonomic analyses were performed using fully grown bacterial cells cultured on R2A agar for 5 days at 30 °C. Except for temperature tests, all other experiments were performed under identical conditions. To determine the optimal growth medium, strains Si-c^T^ and S2-g^T^ were cultured on R2A agar, marine agar (MB Cell), nutrient agar (Difco, Becton, Dickinson and Company, Franklin Lakes, NJ, USA), tryptic soy agar (MB Cell), and Luria–Bertani agar (MB Cell) containing 1.5% agar powder (Duksan, Ansan-si, Republic of Korea). The strains were also grown on R2A agar at different temperatures (4, 10, 15, 20, 25, 28, 30, 35, 37, 40, and 42 °C) and pH levels (pH 1.0–14.0, at pH 1.0 intervals). Salinity tolerance was determined in R2A agar plates supplemented with different concentrations of NaCl (0–5%, at 1.0% intervals). The pH was adjusted using the following buffers before sterilization: hydrochloric acid buffer (pH 1.0–2.0), citrate/NaH_2_PO_4_ buffer (pH 3.0–5.0), phosphate buffer (pH 6.0–8.0), Tris buffer (pH 9.0–10.0), and Na_2_HPO_4_–NaOH buffer (pH 11.0–14.0). Transmission electron microscopy at 80 kV (TEM; JEM1010, JEOL, Akishima, Japan) was performed to assess the cell morphology, as described previously [44]. In brief, the cells were suspended in autoclaved distilled water, placed on a grid, and negatively stained with uranyl acetate for 5–10 s. Motility was assessed by stabbing a needle contaminated with bacterial cells into R2A medium containing 0.4% agar. Anaerobic growth was assessed by culturing the strains on R2A agar at 30 °C for 14 days in an AnaeroPack rectangular system with oxygen absorber strips (Mitsubishi Gas Chemical Company, Tokyo, Japan) to eliminate oxygen. Hydrolytic activities were assessed using R2A agar containing casein (1%, *w*/*v*, skim milk; Biopure, GenomicBase, Namyangju-si, Republic of Korea), carboxymethyl cellulose (CM cellulose, 1%, *w*/*v*; Duksan), chitin (2%, *w*/*v*; Tokyo Chemical Industry Co., Ltd., Tokyo, Japan), and Tween 20 and 80 (1.5%; Samchun, Seoul, Republic of Korea) [45]. DNase activity was confirmed on DNase agar (MB Cell). Gram reactions were assessed using the standard Gram staining method and the non-staining KOH lysis method (3% KOH) [46]. Catalase activity was assessed using a hydrogen peroxide solution (3%, *v*/*v*); the production of bubbles indicated a positive result. Furthermore, oxidase activity was assessed using a tetramethyl-p-phenylenediamine solution (1%, *w*/*v*; bioMérieux, Marcy-l’Étoile, France); a color change to purple indicated a positive result. Enzymatic and metabolic activities were assessed using API 20NE strips (bioMérieux).

### 2.5. Chemotaxonomic Analysis

Fatty acids were extracted through saponification, methylation, and extraction, as described previously [47]. The extracts were identified using the Sherlock Microbial Identification System version 6.3 and RTSBA6 libraries. Polar lipids were extracted and developed using two-dimensional thin-layer chromatography [48]. A mixture of chloroform/methanol/water in a ratio of 65:25:4 (*v*/*v*/*v*) was used in the first dimension, and a mixture of chloroform/methanol/acetic acid/water in a ratio of 80:15:12:4 (*v*/*v*/*v*/*v*) was used in the second dimension. Spots were visualized using the following reagents: 5% molybdophosphoric acid (Sigma-Aldrich, Burlington, MA, USA) for total lipids, 0.2% ninhydrin reagent (Sigma-Aldrich) for amino lipids, α-naphthol reagent for glycolipids, and Zinzadze’s reagent (molybdenum blue spray reagent, 1.3%; Sigma-Aldrich) for phospholipids. Isoprenoid quinones were extracted using a mixture of chloroform and methanol in a ratio of 2:1 (*v*/*v*), as reported previously [49].

### 2.6. Plant Growth-Promoting Traits

#### 2.6.1. Quantification of Indole Acetic Acid (IAA)

IAA was detected and quantified using a previously described method [5]. Strains Si-c^T^ and S2-g^T^ were grown in R2A broth for 3 days at 30 °C with shaking at 180 rpm. The absorbance of the bacterial cells was measured at 600 nm, and the OD at 600 nm was adjusted to 1. Subsequently, 100 μL of the bacterial suspension was inoculated into 10 mL of R2A broth supplemented with 0.1% tryptophan. The medium was prepared by autoclaving the R2A broth, adding tryptophan, and sterilizing the filter.

The inoculated medium was incubated at 30 °C with shaking at 180 rpm for 5 days. The bacterial cells were centrifuged at 10,000 rpm for 5 min, and 1 mL of the bacterial supernatant was then mixed with 2 mL of Salkowski’s reagent (98 mL of 35% perchloric acid and 2 mL of 0.5 M FeCl_3_). The mixture was incubated at 30 °C with shaking at 180 rpm for 30 min, and the absorbance was spectrophotometrically measured at 530 nm. The experiment was performed in triplicate for each strain. IAA production was confirmed by a change in color from yellow to pink. The IAA concentration was determined using an IAA standard curve.

#### 2.6.2. Nitrogen Fixation

The nitrogen-fixing ability of the isolated bacterial strains was confirmed using Jensen’s medium (sucrose 20 g, K_2_HPO_4_ 1 g, MgSO_4_ 0.5 g, NaCl 0.5 g, FeSO_4_ 0.1 g, Na_2_MoO_4_ 0.005 g, CaCO_3_ 2 g, agar 15 g, and distilled water 1 L) [50]. The bacterial strains were inoculated on Jensen’s medium and incubated at 30 °C for one week. Abundant growth on the medium indicated that the strains were capable of fixing atmospheric nitrogen.

#### 2.6.3. Siderophore Production

Isolated strains were grown on chrome azurol sulfonate (CAS) agar plates to assess siderophore production as described previously [50]. Cultures were incubated at 30 °C for one week. Siderophore production was indicated by a color change in the medium around the bacterial colonies, demonstrating the presence of iron-chelating activity.

#### 2.6.4. Phosphate Solubilization

The phosphate solubilization ability of bacterial strains was evaluated using Pikovskaya’s agar [51]. Bacterial strains were inoculated onto the agar plates (yeast extract 0.5 g, glucose 10 g, Ca_3_(PO_4_)_2_ 5 g, (NH_4_)_2_SO_4_ 0.5 g, KCl 0.2 g, MgSO_4_·7H_2_O 0.1 g, MnSO_4_·H_2_O 0.002 g, NaCl 0.2 g, FeSO_4_ 0.002 g, agar 15 g, and distilled water 1 L, pH 7.0) with 0.025 g bromophenol blue. The plates were incubated at 30 °C for one week. Phosphate solubilization was assessed by observing the formation of clear halos around inoculated spots.

#### 2.6.5. Quantification of Ammonia

To quantify ammonia production, 100 uL of bacterial cultures adjusted to OD_600_ = 1.0 was inoculated into 10 mL of peptone water (NaCl 10 g, peptone 5 g, distilled water 1 L) [52]. The inoculum was incubated at 30 °C for 2 days in a shaking incubator set to 180 rpm. After incubation, 0.5 mL of Nessler’s reagent was added to the supernatant of each sample to detect ammonia production. The experiment was conducted in triplicate for each strain. The amount of ammonia was quantified using a standard curve created with ammonium sulfate. Positive results were indicated by a color change to brown or orange, reflecting the presence of ammonia.

## 3. Results and Discussion

### 3.1. Phylogenetic Analysis Based on 16S rRNA Gene Sequences

**Figure 1:**
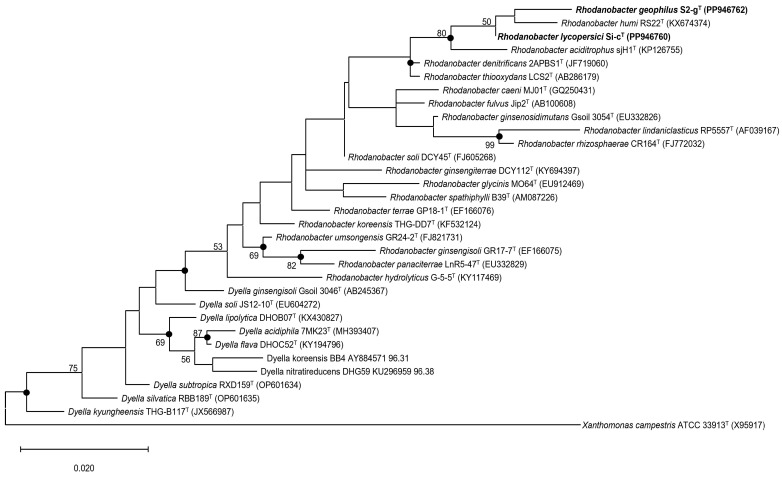
Phylogenetic tree constructed based on 16S rRNA gene sequences using the maximum likelihood algorithm. The tree was rooted using *Xanthomonas campestris* ATCC 33913^T^ (X95917) as an outgroup. The taxonomic relationships between strains Si-c^T^ and S2-g^T^ and other closely related species are depicted. Bootstrap values (>50%) based on 1000 replications are shown at the branch points. Bar, 0.020 substitutions per nucleotide position.

A comparison of the 16S rRNA gene sequences obtained using the Sanger method and the ContEst16S tool revealed 100% identity for both strains (Si-c^T^ and S2-g^T^). The sequence lengths based on the Sanger method and ContEst16S tool were 1465 and 1541 bp for strain Si-c^T^ and 1468 and 1541 bp for strain S2-g^T^, respectively. The similarity between strains Si-c^T^ and S2-g^T^ was 99.0%. Comparative analysis of 16S rRNA gene sequences using the EzBioCloud server revealed that strain Si-c^T^ exhibited the highest similarity to *R*. *humi* RS22^T^ (99.1%), *R*. *denitrificans* 2APBS1^T^ (98.4%), and *R*. *thiooxydans* LCS2^T^ (98.2%), while strain S2-g^T^ exhibited the highest similarity to *R*. *humi* RS22^T^ (98.6%), *R*. *aciditrophus* sjH1^T^ (98.0%), and *R*. *denitrificans* 2APBS1^T^ (97.7%). The phylogenetic trees based on the 16S rRNA gene sequences revealed that strains Si-c^T^ and S2-g^T^ clustered well with species belonging to the genus *Rhodanobacter* (Figure 1 and Figure S1).

Notably, in all trees, the two novel bacterial strains exhibited a close relationship with *R*. *humi* RS22^T^ and *R*. *aciditrophus* sjH1^T^. Strains Si-c^T^ and S2-g^T^ clustered effectively with members of the genus *Rhodanobacter* and formed a distinct lineage with their closest relatives. Based on consistent genealogical evidence, these strains should be classified as novel species within the genus *Rhodanobacter*.

### 3.2. Genomic Analysis and Genome Annotation

The genome of strain Si-c^T^ had a total size of 4320329 bp with a G + C content of 67.0 mol%. It consisted of 15 contigs, 3821 genes, 3734 protein-coding genes, 47 tRNAs, three rRNAs, and 33 total pseudogenes. The genome coverage was 146.3×. The 16S rRNA gene sequences obtained from the genome were 1541 bp in length and matched 100% with those obtained using the Sanger sequencing method.

The genome of strain S2-g^T^ had a total size of 3752455 bp with a G + C content of 68.5 mol%. It consisted of 31 contigs, 3376 genes, 3281 protein-coding genes, 47 tRNAs, three rRNAs, and 41 total pseudogenes. The genome coverage was 145.8×. The 16S rRNA gene sequences obtained from the genome were 1541 bp in length and matched 100% with those obtained using the Sanger sequencing method.

**Figure 2:**
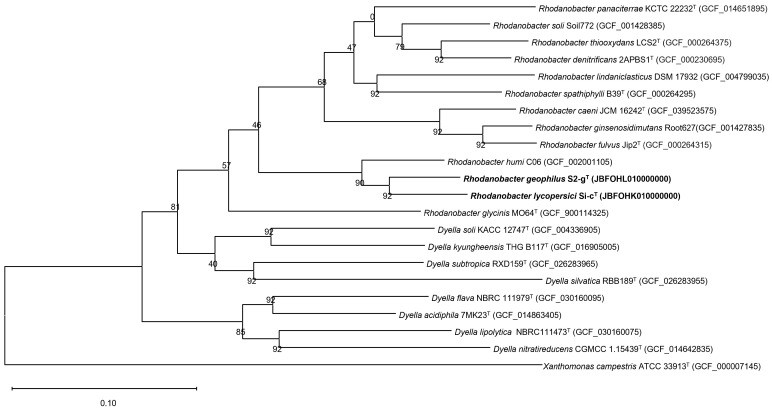
Phylogenetic tree using a set of 92 bacterial core genes. *Xanthomonas campestris* ATCC 33913^T^ was selected as an outgroup. The numbers at the nodes represent the gene support index, with the maximum value being 92. Bar, 0.10 substitutions per nucleotide position.

The completeness and contamination values were 99.9% and 1.7% for strain Si-c^T^ and 99.9% and 0.1% for strain S2-g^T^, respectively, as affirmed by CheckM results. Phylogenomic trees constructed based on the UBCG and AAI revealed that the two isolated strains were positioned within the genus *Rhodanobacter* (Figure 2 and Figure S2); this finding is consistent with the results obtained from the 16S rRNA-based trees.

Through the NCBI annotation, several genes were identified that contribute to motility, pH tolerance, and plant growth-promoting traits. Motility-related genes such as *flg*, *flh*, *fli*, and *motD* were detected, which are known to contribute to flagellar assembly. Notably, the *fliL* gene, found in both isolates but absent in the reference strains, was also identified. Flagella-related genes for isolated strains and the reference strains are presented in Table S1.

Additionally, several genes related to pH tolerance were identified, including *atp*, *pho*, *cyo*, *cco*, *clp*, and *pst*. These genes play critical roles in energy metabolism, phosphate transport, and stress responses, helping the bacteria adapt to varying pH conditions (Table S2). In terms of plant growth-promoting traits, genes associated with IAA production, such as *trpABCDE*, were found. Siderophore production was supported by the detection of two genes, a siderophore-interacting protein, and the catecholate siderophore receptor Fiu, both involved in siderophore synthesis and transport [53]. Phosphate solubilization-related genes, including *ppk*, *pho*, and *pst*, and PQQ-dependent enzymes were detected. Specifically, *ppk* contributes to polyphosphate synthesis, enabling phosphate storage and utilization, while *pho* and *pst* are involved in phosphate transport and regulation, allowing phosphate acquisition under limiting conditions [54]. Additionally, PQQ-dependent enzymes enhance phosphate solubilization by producing organic acids, which lower the pH and convert insoluble phosphates into soluble forms usable by plants [55]. These genes indicate a strong potential for phosphate solubilization, improving phosphorus availability in soil environments. Ammonia production genes such as *glnA*, *glnE*, *hutH*, and *ilvA* were also identified. *glnA* and *glnE* are involved in nitrogen assimilation and ammonia synthesis, while *hutH* and *ilvA* contribute to ammonia production through histidine and threonine degradation, respectively. These genes underscore the strains’ strong ammonia production capabilities, enhancing nitrogen availability for plant growth [56]. Although no nitrogen fixation genes were identified in either strain, several nitrogen metabolism-related genes were detected. Both strains contained *glnK*, *ntrC*, and *gltB*, which are involved in nitrogen regulation and assimilation. In strain Si-c^T^, additional genes such as *narHIJ* and *nirK* were found, facilitating nitrate reduction and denitrification processes. Other relevant genes, including nitric-oxide reductase, nitrilase-related carbon–nitrogen hydrolase, and nitrate/nitrite transporter, highlighted the ability to metabolize nitrogen efficiently. These pathways enabled the strains to convert nitrate into ammonia and assimilate it, allowing for limited growth even in the absence of fixed nitrogen sources by facilitating ammonia assimilation and recycling through nitrate reduction and nitrogen assimilation mechanisms [57]. The genes related to plant growth-promoting traits are listed in Table 1.

RAST analysis revealed that strains Si-c^T^ and S2-g^T^ had the highest number of genes in the following categories in the given order: amino acids and derivatives, protein metabolism and cofactors, vitamins, prosthetic groups, and pigments. This order aligns with that of other closely related species. Specifically, strain Si-c^T^ had 230, 182, and 158 genes associated with these categories, while strain S2-g^T^ had 234, 178, and 144 genes in these categories, respectively. Species with a high number of genes in these categories likely exhibit efficient metabolic pathways. Details of the RAST analysis are provided in Table S3. A set of tools integrated into the Proksee online server was used for comprehensive genome annotation and visualization of the circular genomic map (Figure S3). Prokka pipeline analysis revealed that strain Si-c^T^ contained a total of 3771 features, including 3716 CDSs, 51 tRNAs, three rRNAs, and one tmRNA. Moreover, it contained 11 CRISPRs, two CARDs, and 79 genes predicted using Alien Hunter. MobileOG-db revealed 91 genomic features related to the replication/recombination/repair, integration/excision, phase, transfer, and stability/transfer/defense of mobile genetic elements. For strain S2-g^T^, Prokka pipeline analysis revealed a total of 3344 features, including 3289 CDSs, 55 tRNAs, three rRNAs, and one tmRNA. This strain also contained seven CRISPRs, two CARDs, and 99 genes predicted using Alien Hunter. MobileOG-db revealed a total of 73 genomic features associated with the replication/recombination/repair, integration/excision, phase, transfer, and stability/transfer/defense of mobile genetic elements. Moreover, analysis using OrthoVenn 3.0 software revealed a total of 4012 OCs, including 2103 core OCs shared by all strains, 1926 accessory OCs shared by more than two but not all strains, and 73 unique OCs. A total of 24 OCs were unique to strain Si-c^T^, while 8 OCs were unique to strain S2-g^T^. These findings highlight the common genetic features between these strains and the distinct genetic traits that differentiate strains Si-c^T^ and S2-g^T^ (Figure S4). Using antiSMASH 7.1.0, several BGCs were predicted in the genomes of strains Si-c^T^ and S2-g^T^. Six BGCs were identified in strain Si-c^T^, and seven were identified in strain S2-g^T^. The genome of strain Si-c^T^ contained the following BGCs: aryl polyene (BGCs 1 and 3), terpene (BGC 2), RiPP-like (BGC 4), NRPS, T1PKS (BGC 5), and lasso peptide (BGC 6). On the other hand, the genome of strain S2-g^T^ contained the following BGCs: aryl polyene (BGCs 1 and 2), terpene (BGC 3), T1PKS, NRPS-like, NRPS (BGC 4), NRPS, NRPS-like (BGC 5), lasso peptide (BGC 6), and NRPS (BGC 7). The BGC types identified in the genomes of these two strains exhibited notable similarities, including the presence of aryl polyene, terpene, T1PKS, NRPS, and lasso peptide BGCs. Both strains shared these BGC types, suggesting similar biosynthetic capabilities. However, strain Si-c^T^ contained an additional RiPP-like BGC that was not found in strain S2-g^T^, while strain S2-g^T^ contained NRPS-like BGCs that were absent in strain Si-c^T^. Thus, while the two strains have a similar core set of BGCs, there are distinct differences in their biosynthetic potential. Among all BGCs in the novel strains, only one BGC exhibited more than 50% similarity to a known BGC. It was 100% identical to a rhizomide A BGC from *Paraburkholderia rhizoxinica* HKI 454^T^ (NC_014718.1). The other BGCs showed only minimal or no similarity to previously identified BGCs, suggesting that the novel strains Si-c^T^ and S2-g^T^ have significant potential for producing new natural products. Details regarding the BGCs present in the genomes of the two novel strains and their functions are presented in Table S4. The calculated OGRI for strain Si-c^T^ ranged from 75.4% to 89.5% for AAI, 79.2% to 89.4% for ANI, and 22.4% to 37.3% for dDDH. For strain S2-g^T^, the calculated OGRI ranged from 75.5% to 89.5% for AAI, 79.5% to 89.4% for ANI, and 22.5% to 37.3% for dDDH. All these values met the recognized cutoff thresholds for distinguishing bacterial species (95–96% for ANI, 95%–96% for AAI, and 70% for dDDH) [58,59,60]. The OGRI values between the two novel bacterial strains and recognized *Rhodanobacter* species are presented in Table 2.

### 3.3. Physiological and Chemotaxonomic Analysis

Cells of strains Si-c^T^ and S2-g^T^ were Gram-negative, oxidase-positive, aerobic, and motile by means of flagella. They had a rod-shaped morphology (Figure S5) and formed visible yellow colonies on R2A agar at 30 °C within 3 days. Strain Si-c^T^ was catalase-positive, whereas strain S2-g^T^ was catalase-negative. Flagella were detected by TEM. The differential physiological and biochemical features of the two novel strains compared with other closely related species within the genus *Rhodanobacter* are presented in Table 3.

Both strains exhibited vigorous growth on R2A agar, nutrient agar, and tryptic soy agar; slight growth on marine agar; and no growth on Luria–Bertani agar. On R2A media, strain Si-c^T^ grew at temperatures of 10–37 °C (optimum, 28–30 °C), at pH levels of 3.0–13.0 (optimum, pH 6.0–7.0), and in the presence of up to 4% NaCl (optimum, 0%), while strain S2-g^T^ grew at temperatures of 10–42 °C (optimum, 28–30 °C), at pH levels of 2.0–13.0 (optimum, pH 7.0–8.0), and in the presence of up to 4% NaCl (optimum, 0%). Strain S2-g^T^ hydrolyzed casein and CM cellulose; however, strain Si-c^T^ did not hydrolyze any of the tested substrates. In the API 20NE tests, both strains exhibited positive results for esculin hydrolysis; *β*-galactosidase activity; and _D_-glucose, _D_-mannose, *N*-acetylglucosamine, and _D_-maltose assimilation but negative results for indole production; glucose fermentation; _L_-arginine hydrolysis; urease activity; gelatin hydrolysis; and _L_-arabinose, _D_-mannitol, potassium gluconate, capric acid, adipic acid, malate, trisodium citrate, and phenylacetic acid assimilation.

The major fatty acids in strains Si-c^T^ and S2-g^T^ were iso-C_15:0_ (17.3% and 18.6%, respectively), iso-C_16:0_ (13.6% and 9.6%, respectively), iso-C_17:0_ (15.8% and 10.6%, respectively), and summed feature 9 (iso-C_17:1_ *ω*9*c* and/or C_16:0_ *ω*6*c* 10-methyl; 19.4% and 21.3%, respectively). The fatty acid profiles of strains Si-c^T^ and S2-g^T^ were generally consistent with those of closely related *Rhodanobacter* species, although slight deviations were noted in detailed proportions. Both strains exhibited lower proportions of iso-C_15:0_ and summed feature 9 than the reference strains. They also exhibited lower proportions of summed feature 3 (C_16:1_ *ω*7*c* and/or C_16:1_ ω6*c*; 2% in strain Si-c^T^ and 3.2% in strain S2-g^T^). The detailed fatty acid profiles are presented in Table S5.

The polar lipid profiles of both strains consisted of phosphatidylethanolamine, diphosphatidylglycerol, phosphatidyl-*N*-methylethanolamine, and phosphatidylglycerol, which are commonly reported as polar lipids in the genus *Rhodanobacter*. However, strain Si-c^T^ was distinguished from recognized *Rhodanobacter* species by the presence of five unknown aminophosphoglycolipids, two unknown aminophospholipids, two unknown phosphoglycolipids, two unknown glycolipids, and three unknown phospholipids, in addition to their respective positions on the chromatogram. Compared with strain Si-c^T^, strain S2-g^T^ also contained an additional unknown phosphoglycolipid and two unknown glycolipids, with differences being observed in the precise positions of each spot on the chromatogram (Figure S6).

### 3.4. Plant Growth-Promoting Traits

**Figure 3:**
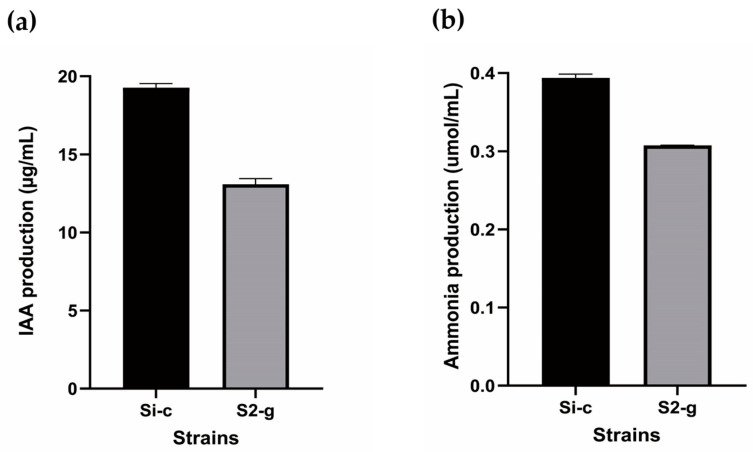
Quantification of IAA (**a**) and ammonia (**b**) of strains Si-c^T^ and S2-g^T^. The values represent the mean and standard deviation from three replicates.

According to Salkowski’s test, strain Si-c^T^ produced 19.3 µg/mL of IAA, which was greater than the 13.1 µg/mL produced by strain S2-g^T^ (Figure 3a). These levels were comparable with the IAA production of the PGPB strain *Pseudomonas azotoformans* NBRC 12693^T^ [64]. In the ammonia production assay, Si-c^T^ produced 0.39 µmol/mL, while S2-g^T^ produced 0.31 µmol/mL (Figure 3b), values comparable to those of the PGPB strain *Enterobacter hormaechei* J146 [65].

**Figure 4:**
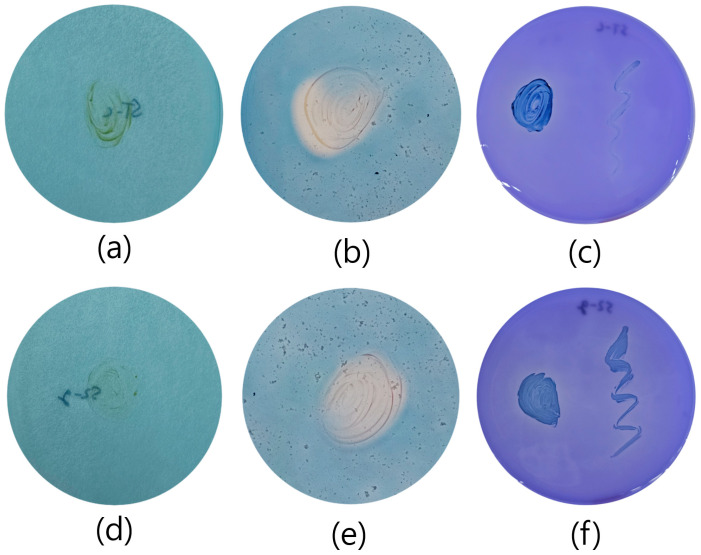
Results of the plant growth-promoting traits, including nitrogen fixation, siderophore production, and phosphate solubilization. Strains Si-c^T^ and S2-g^T^ are shown in (**a**–**f**), respectively. (**a**,**d**) represent nitrogen fixation; (**b**,**e**) represent siderophore production; (**c**,**f**) represent phosphate solubilization.

Both strains exhibited weak growth on the Jensen medium; however, since nitrogen fixation genes were not detected, this growth cannot be attributed to nitrogen fixation. For siderophore production and phosphate solubilization, clear zones were observed around both strains, confirming their capabilities (Figure 4).

The two isolated strains possess various plant growth-promoting traits and related genes, and their ability to survive in extreme pH environments indicates their potential as valuable contributions to plant growth. However, since only in vitro experiments have been conducted, further validation in practical applications is needed.

## 4. Conclusions

This study identified two novel bacterial species, *Rhodanobacter lycopersici* sp. nov. and *Rhodanobacter geophilus* sp. nov., isolated from the rhizosphere of *Solanum lycopersicum*. Both strains exhibited broad pH tolerance and key plant growth-promoting traits, including IAA production, siderophore production, ammonia production, and phosphate solubilization.

The discovery of these novel species not only broadens our understanding of the genus *Rhodanobacter* but also offers promising potential for their application in sustainable agriculture, particularly in soils with fluctuating pH levels. While this study was limited to in vitro experiments, further pot experiments will provide valuable insights into the practical agricultural applications of these strains. The isolation and characterization of these two novel strains provide valuable insights into their functional traits and provide a solid starting point for future studies focused on promoting plant growth and soil health under diverse environmental conditions.

### 4.1. Description of Rhodanobacter lycopersici sp. nov.

*R*. *lycopersici* (ly.co.per’si.ci. N.L. gen. n. *lycopersici*, of the plant genus *Lycopersicon*).

Cells are Gram-negative, aerobic, motile, oxidase-positive, and catalase-positive. On TEM analysis, the cells appear rod-shaped and flagellated, measuring 1.0–1.1 µm in length and 0.5–0.6 µm in width. Colonies on R2A agar are pale yellow, raised, opaque with a translucent edge, entire, smooth, and circular. Growth is abundant on R2A agar, nutrient agar, and tryptic soy agar; weak on marine agar; and absent on Luria–Bertani agar. The growth conditions are as follows: temperature, 15–37 °C (optimum, 28–30 °C); pH, 3.0–13.0 (optimum, pH 6.0–7.0); and NaCl concentration, 0–4.0% (*w*/*v*; optimum, 0%). The results of hydrolysis tests are negative. In the API 20NE tests, the cells are positive for the reduction of nitrate; hydrolysis of esculin and *β*-galactosidase; and assimilation of _D_-glucose, _D_-mannose, *N*-acetylglucosamine, and _D_-maltose; all other results are negative. The predominant cellular fatty acid profiles are iso-C_15:0_, iso-C_16:0_, iso-C_17:0_, and summed feature 9 (comprising iso-C_17:1_ *ω*9*c* and/or C_16:0_ *ω*6*c* 10-methyl). Q-8 is the predominant respiratory quinone. The polar lipid profiles consist of phosphatidylethanolamine, diphosphatidylglycerol, phosphatidyl-N-methylethanolamine, phosphatidylglycerol, five unknown aminophosphoglycolipids, two unknown aminophospholipids, two unknown phosphoglycolipids, two unknown glycolipids, and three unknown phospholipids.

The type strain Si-c^T^ (=KACC 23732^T^ = TBRC 19125^T^) was isolated from the rhizosphere of a tomato plant in Ilsan, Republic of Korea.

### 4.2. Description of Rhodanobacter geophilus sp. nov.

*R*. *geophilus* (ge.o’phi.lus. Gr. n. *ge*, earth; Gr. suff. -*philos*, loving; N.L. masc. adj. *geophilus*, earth loving).

Cells are Gram-negative, aerobic, motile, oxidase-positive, and catalase-negative. On TEM analysis, the cells appear rod-shaped and flagellated, measuring 1.2–1.6 µm in length and 0.4–0.5 µm in width. Colonies on R2A agar are initially yellow and gradually turn pale brown. The colonies appear raised, opaque with a translucent edge, entire, smooth, and circular. Growth is abundant on R2A agar, nutrient agar, and tryptic soy agar; weak on marine agar; and absent on Luria–Bertani agar. The growth conditions are as follows: temperature, 15–42 °C (optimum, 28–30 °C); pH, 2.0–13.0 (optimum, pH 7.0–8.0); and NaCl concentration, 0–4% (*w*/*v*; optimum, 0%). The results of hydrolysis tests are all negative. In the API 20NE tests, the cells are positive for the hydrolysis of esculin and *β*-galactosidase and assimilation of _D_-glucose, _D_-mannose, *N*-acetylglucosamine, and _D_-maltose; all other results are negative. The predominant cellular fatty acid profiles are iso-C15:0, iso-C_17:0_, and summed feature 9 (comprising iso-C_17:1_ *ω*9*c* and/or C_16:1_ *ω*6*c* 10-methyl). Q-8 is the predominant respiratory quinone. The polar lipid profiles consist of phosphatidylethanolamine, diphosphatidylglycerol, phosphatidyl-N-methylethanolamine, phosphatidylglycerol, five unknown aminophosphoglycolipids, two unknown aminophospholipids, three unknown phosphoglycolipids, four unknown glycolipids, and three unknown phospholipids.

The type strain S2-g^T^ (=KACC 23733^T^ = TBRC 19012^T^) was isolated from the rhizosphere of a tomato plant in Ilsan, Republic of Korea.

## Figures and Tables

**Table 1:** Plant growth-promoting traits-related genes identified in strains (A) Si-c^T^ and (B) S2-g^T^. The table lists genes involved in IAA production, nitrogen metabolism, siderophore production, phosphate solubilization, and ammonia production. These genes were annotated using NCBI annotation platform. +, the forward transcription strand; −, the reverse transcription strand.

**(A) Si-c^T^**							
**Traits**	**Gene**	**Protein Name**	**Accession**	**Start**	**Stop**	**Strand**	**Length (aa)**
IAA Production	*trpA*	Tryptophan synthase subunit alpha	JBFOHK010000001	582,844	583,644	−	266
	*trpB*	Tryptophan synthase subunit beta	JBFOHK010000001	583,641	584,852	−	403
	*trpE*	Anthranilate synthase component I	JBFOHK010000002	726,143	727,618	+	491
	*trpD*	Anthranilate phosphoribosyltransferase	JBFOHK010000002	748,695	749,750	+	351
	*trpC*	Indole-3-glycerol phosphate synthase TrpC	JBFOHK010000002	749,819	750,613	+	264
		Tryptophan--tRNA ligase	JBFOHK010000001	1,478,563	1,479,909	+	448
		Tryptophan 2,3-dioxygenase	JBFOHK010000001	1,487,847	1,488,695	−	282
		Phosphoribosylanthranilate isomerase	JBFOHK010000001	586,128	586,745	−	205
Nitrogen Metabolism	*narH*	Nitrate reductase subunit beta	JBFOHK010000004	23,785	25,326	−	513
	*narI*	Respiratory nitrate reductase subunit gamma	JBFOHK010000004	22,432	23,139	−	235
	*narJ*	Nitrate reductase molybdenum cofactor assembly chaperone	JBFOHK010000004	23,168	23,785	−	205
	*nirK*	Copper-containing nitrite reductase	JBFOHK010000001	244,694	246,220	+	508
		Nitric-oxide reductase large subunit	JBFOHK010000001	239,631	241,928	−	765
		Nitrilase-related carbon–nitrogen hydrolase	JBFOHK010000003	308,876	310,303	+	475
		Nitrate/nitrite transporter	JBFOHK010000004	29,107	30,357	−	416
	*glnK*	P-II family nitrogen regulator	JBFOHK010000002	417,051	417,389	−	112
		P-II family nitrogen regulator	JBFOHK010000004	437,822	438,160	+	112
		Carbon–nitrogen hydrolase	JBFOHK010000001	189,566	190,456	−	296
	*ntrC*	Nitrogen regulation protein NR(I)	JBFOHK010000002	88,709	90,127	−	472
		Nitrogen regulation protein NR(II)	JBFOHK010000002	90,124	91,131	−	335
	*gltB*	Glutamate synthase large subunit	JBFOHK010000005	185,142	189,584	+	1480
		FMN-binding glutamate synthase family protein	JBFOHK010000001	1,075,646	1,077,154	−	502
Siderophore Production		Siderophore-interacting protein	JBFOHK010000001	740,009	740,797	+	262
		Catecholate siderophore receptor Fiu	JBFOHK010000004	298,978	301,326	+	782
Phosphate Solubilization		PQQ-dependent sugar dehydrogenase	JBFOHK010000001	290,539	291,759	−	406
		PQQ-binding-like beta-propeller repeat protein	JBFOHK010000001	448,189	449,700	−	503
	*ppk1*	Polyphosphate kinase 1	JBFOHK010000004	105,024	107,168	+	714
	*ppk1*	Polyphosphate kinase 1	JBFOHK010000004	423,147	425,255	+	702
	*ppk2*	Polyphosphate kinase 2	JBFOHK010000001	1,336,814	1,337,578	+	254
	*phoB*	Phosphate regulon transcriptional regulator PhoB	JBFOHK010000004	421,015	421,704	+	229
	*phoR*	Phosphate regulon sensor histidine kinase PhoR	JBFOHK010000004	422,042	423,091	+	349
	*phoU*	Phosphate signaling complex protein PhoU	JBFOHK010000001	638,194	638,925	+	243
	*pstA*	Phosphate ABC transporter permease PstA	JBFOHK010000001	636,381	637,250	+	289
	*pstB*	Phosphate ABC transporter ATP-binding protein PstB	JBFOHK010000001	637,243	638,058	+	271
	*pstC*	Phosphate ABC transporter permease subunit PstC	JBFOHK010000001	635,410	636,381	+	323
	*pstS*	Phosphate ABC transporter substrate-binding protein PstS	JBFOHK010000001	634,202	635,218	+	338
Ammonia Production	*glnA*	Type I glutamate--ammonia ligase	JBFOHK010000002	96,336	97,745	−	469
	*glnE*	Bifunctional [glutamate--ammonia ligase]-adenylyl-L-tyrosine phosphorylase/[glutamate--ammonia-ligase] adenylyltransferase	JBFOHK010000005	351,827	354,676	+	949
		Ammonium transporter	JBFOHK010000004	438,147	439,490	+	447
		L-serine ammonia-lyase	JBFOHK010000002	861,277	862,659	−	460
	*hutH*	Histidine ammonia-lyase	JBFOHK010000003	215,896	217,797	−	633
	*ilvA*	Threonine ammonia-lyase, biosynthetic	JBFOHK010000005	59,564	61,132	−	522
**(B) S2-g^T^**							
**Traits**	**Gene**	**Protein Name**	**Accession**	**Start**	**Stop**	**Strand**	**Length (aa)**
IAA Production	*trpB*	Tryptophan synthase subunit beta	JBFOHL010000002	114,807	116,018	+	403
	*trpA*	Tryptophan synthase subunit alpha	JBFOHL010000002	116,015	116,815	+	266
	*trpC*	Indole-3-glycerol phosphate synthase TrpC	JBFOHL010000018	31,276	32,070	−	264
	*trpD*	Anthranilate phosphoribosyltransferase	JBFOHL010000018	32,135	33,184	−	349
	*trpE*	Anthranilate synthase component I	JBFOHL010000018	49,841	51,316	−	491
		Tryptophan--tRNA ligase	JBFOHL010000019	4284	5630	+	448
		Tryptophan 2,3-dioxygenase	JBFOHL010000019	12,868	13,716	−	282
		Phosphoribosylanthranilate isomerase	JBFOHL010000002	112,135	112,770	+	211
Nitrogen Metabolism	*glnK*	P-II family nitrogen regulator	JBFOHL010000001	297,354	297,692	+	112
		P-II family nitrogen regulator	JBFOHL010000012	49,216	49,554	−	112
		Carbon–nitrogen hydrolase	JBFOHL010000017	32,018	32,908	−	296
	*ntrC*	Nitrogen regulation protein NR(I)	JBFOHL010000011	96,362	97,741	+	459
		Nitrogen regulation protein NR(II)	JBFOHL010000011	95,248	96,255	+	335
	*gltB*	Glutamate synthase large subunit	JBFOHL010000008	141,185	145,627	−	1480
		FMN-binding glutamate synthase family protein	JBFOHL010000006	52,390	53,901	−	503
Siderophore Production		Catecholate siderophore receptor Fiu	JBFOHL010000003	276,284	278,641	+	785
		Siderophore-interacting protein	JBFOHL010000004	8785	9567	+	260
Phosphate Solubilization		PQQ-binding-like beta-propeller repeat protein	JBFOHL010000002	269,737	270,987	+	416
		PQQ-binding-like beta-propeller repeat protein	JBFOHL010000004	101,633	102,091	−	152
		PQQ-dependent sugar dehydrogenase	JBFOHL010000002	386,884	388,104	−	406
	*ppk1*	Polyphosphate kinase 1	JBFOHL010000003	117,749	119,893	+	714
	*ppk1*	Polyphosphate kinase 1	JBFOHL010000012	60,438	62,531	−	697
	*phoB*	Phosphate regulon transcriptional regulator PhoB	JBFOHL010000012	65,413	66,102	−	229
	*phoR*	Phosphate regulon sensor histidine kinase PhoR	JBFOHL010000012	64,073	65,371	−	432
	*phoU*	Phosphate signaling complex protein PhoU	JBFOHL010000012	71,639	72,370	−	243
	*pstA*	Phosphate ABC transporter permease PstA	JBFOHL010000002	73,333	74,202	−	289
	*pstB*	Phosphate ABC transporter ATP-binding protein PstB	JBFOHL010000002	72,513	73,340	−	275
	*pstC*	Phosphate ABC transporter permease subunit PstC	JBFOHL010000002	74,202	75,173	−	323
	*pstS*	Phosphate ABC transporter substrate-binding protein PstS	JBFOHL010000002	75,395	76,429	−	344
Ammonia Production	*glnA*	Type I glutamate--ammonia ligase	JBFOHL010000011	88,689	90,098	+	469
	*glnE*	Bifunctional [glutamate--ammonia ligase]-adenylyl-L-tyrosine phosphorylase/[glutamate--ammonia-ligase] adenylyltransferase	JBFOHL010000015	31,987	34,836	+	949
		Ammonium transporter	JBFOHL010000012	47,901	49,226	−	441
		L-serine ammonia-lyase	JBFOHL010000016	57,527	58,909	−	460
	*hutH*	Histidine ammonia-lyase	JBFOHL010000009	170,273	172,174	−	633
	*ilvA*	Threonine ammonia-lyase, biosynthetic	JBFOHL010000013	65,891	67,462	−	523

**Table 2:** Overall genome relatedness index (OGRI) between strains Si-c^T^ and S2-g^T^ and other *Rhodanobacter* species (AAI, ANI, and dDDH values).

	Si-c^T^			S2-g^T^		
	AAI (%)	ANI (%)	dDDH (%)	AAI (%)	ANI (%)	dDDH (%)
*R. lycopersici* Si-c^T^	100	100	100	89.5	89.4	37.3
*R. geophilus* S2-g^T^	89.5	89.4	37.3	100	100	100
*R. caeni* MJ01^T^	75.6	80.0	23.1	75.8	80.2	23.1
*R. denitrificans* 2APBS1^T^	77.0	81.4	24.1	77.4	81.8	24.6
*R. fulvus* Jip2^T^	75.5	79.2	22.5	75.6	79.6	22.7
*R. ginsenosidimutans* Root627	75.4	79.2	22.4	75.5	79.5	22.5
*R. glycinis* MO64^T^	77.9	80.7	23.7	77.7	80.9	23.7
*R. humi* C06	87.9	88.0	33.9	88.6	88.8	35.6
*R. lindaniclasticus* DSM 17932	75.8	80.2	23.4	76.1	80.6	23.5
*R. panaciterrae* KCTC 22232^T^	76.8	79.5	22.5	76.8	79.7	22.6
*R. soli* JCM 16126^T^	77.3	80.9	23.5	77.4	81.2	23.8
*R. spathiphylli* B39^T^	76.2	80.3	23.2	76.3	80.7	23.4
*R. thiooxydans* LCS2^T^	77.2	81.2	24.3	77.5	81.8	24.7

**Table 3:** Phenotypic features of strains Si-c^T^, S2-g^T^, and type strains of closely related species belonging to the genus *Rhodanobacter*. Strains: 1, Si-c^T^ (data from this study); 2, S2-g^T^ (data from this study); 3, *R*. *humi* RS22^T^ [61]; 4, *R*. *denitrificans* 2APBS1^T^ [62]; and 5, *R*. *thiooxydans* LCS2^T^ [63]. All strains exhibited positive results for oxidase activity; esculin hydrolysis; *β*-galactosidase activity; and _D_-glucose, *N*-acetylglucosamine, and _D_-maltose assimilation but negative results for indole production; arginine hydrolyze and urease activities; and _L_-arabinose, _D_-mannitol, potassium gluconate, capric acid, adipic acid, malate, trisodium citrate, and phenylacetic acid assimilation. +, positive; −, negative; w, weakly positive; ND, no data.

Characteristic	1	2	3	4	5
Growth temperature (°C)	10–37	10–42	15–35	10–35	10–40
pH range	3.0–13.0	2.0–13.0	4.5–11.0	4.0–8.0	5.0–8.0
Salt tolerance (%, *w*/*v*)	0–4	0–4	0–4	0–2	0–2
Catalase	+	−	+	+	+
Motility	+	+	−	+	−
Length	1–1.1	1.2–1.6	1.8–2.9	3.0–5.0	1.5–3.0
Width	0.5–0.6	0.4–0.5	0.8–1.2	0.3–0.5	0.6–0.8
Casein	+	−	−	+	−
Chitin	−	−	−	ND	ND
CM cellulose	+	−	−	ND	ND
DNA	−	−	+	−	w
Tween 20	−	−	ND	ND	ND
Tween 80	−	−	−	ND	ND
Nitrate reduction	+	−	−	+	*+*
Glucose fermentation	−	−	+	−	−
Gelatin hydrolysis	−	−	−	+	+
_D_-Mannose	+	w	−	−	−

## Data Availability

The DDBJ/ENA/GenBank accession numbers for the Whole Genome Shotgun project, 16S rRNA gene sequence obtained using the Sanger method, and 16S rRNA gene sequence obtained from the whole genome are as follows: Si-c^T^: JBFOHK000000000 (Whole Genome Shotgun), PP647361 (Sanger method 16S rRNA), and PP946760 (whole genome 16S rRNA). S2-g^T^: JBFOHL000000000 (Whole Genome Shotgun), PP647365 (Sanger method 16S rRNA), and PP946762 (whole genome 16S rRNA).

## Supplementary Materials

The following supporting information can be downloaded at https://www.mdpi.com/article/10.3390/microorganisms12112227/s1, Figure S1: Phylogenetic trees constructed using 16S rRNA gene sequences with (a) NJ and (b) MP algorithms; Figure S2: AAI tree generated using EzAAI illustrating the phylogenetic positions of the strains Si-c^T^, S2-g^T^, and their closely related species; Figure S3: Graphical circular genomic map generated using the Proksee online server, displaying the genome views for strains (a) Si-c^T^ and (b) S2-g^T^; Figure S4: Venn diagrams built using the OrthoVenn3 online server, exhibiting the number of shared and unique orthologous gene clusters of strains Si-c^T^, S2-g^T^, and other closely related species; Figure S5: Cell morphology under the transmission electron microscope for (a) Si-c^T^ and (b) S2-g^T^; Figure S6: Polar lipid profiles of strains (a) Si-c^T^ and (b) S2-g^T^; Table S1: Overview of flagella-related genes in the genomes of strains Si-c^T^, S2-g^T^, and closely related species within the genus *Rhodanobacter*; Table S2: Genes related to pH tolerance of strains (a) Si-c^T^ and (b) S2-g^T^ identified with NCBI annotation systems; Table S3: Comparison of subsystem category distribution and feature counts for strains Si-c^T^, S2-g^T^, and other closely related taxa using RAST analysis; Table S4: Overview of identified BGCs in the (a) Si-c^T^ and (b) S2-g^T^ genome predicted by antiSMASH; Table S5: Fatty acid compositions (% of the total) of strains Si-c^T^, S2-g^T^, and closely related taxa.
