# Supplementary material for: Two Novel Bacterial Species, Rhodanobacter lycopersici sp. nov. and Rhodanobacter geophilus sp. nov., Isolated from the Rhizosphere of Solanum lycopersicum with Plant Growth-Promoting Traits
Source: Microorganisms. 2024 Nov 3;12(11):2227. doi: 10.3390/microorganisms12112227 (PMC11596576; doi:10.3390/microorganisms12112227)
Supplement: Supplementary file 1 [file microorganisms-12-02227-s001.zip › microorganisms-3272197-supplementary.pdf]

Supplementary Material

Two Novel Bacterial Species, *Rhodanobacter lycopersici* sp. nov. and *Rhodanobacter geophilus* sp. nov., Isolated from the Rhizosphere of *Solanum lycopersicum* with Plant Growth-Promoting Traits

Haejin Woo, Inhyup Kim, Geeta Chhetri, Sunho Park, Hyunji Lee, Subin Yook and Taegun Seo *

Department of Life Science, Dongguk University, Goyang 10326, Republic of Korea

***Corresponding author:**

Taegun Seo

Tel.: +82-31-961-5135

Fax: +82-31-961-5348

E-mail: tseo@dongguk.edu

Figure S1. Phylogenetic trees constructed using 16S rRNA gene sequences with (a) NJ and (b) MP algorithms. Bootstrap values were calculated with 1000 replications, and only above 50% values are shown. Bar represents 0.010 substitutions per nucleotide position for the NJ tree and 20 substitutions per nucleotide position for the MP tree. *Xanthomonas campestris* ATCC 33913^T was selected as an outgroup.

(a) NJ tree

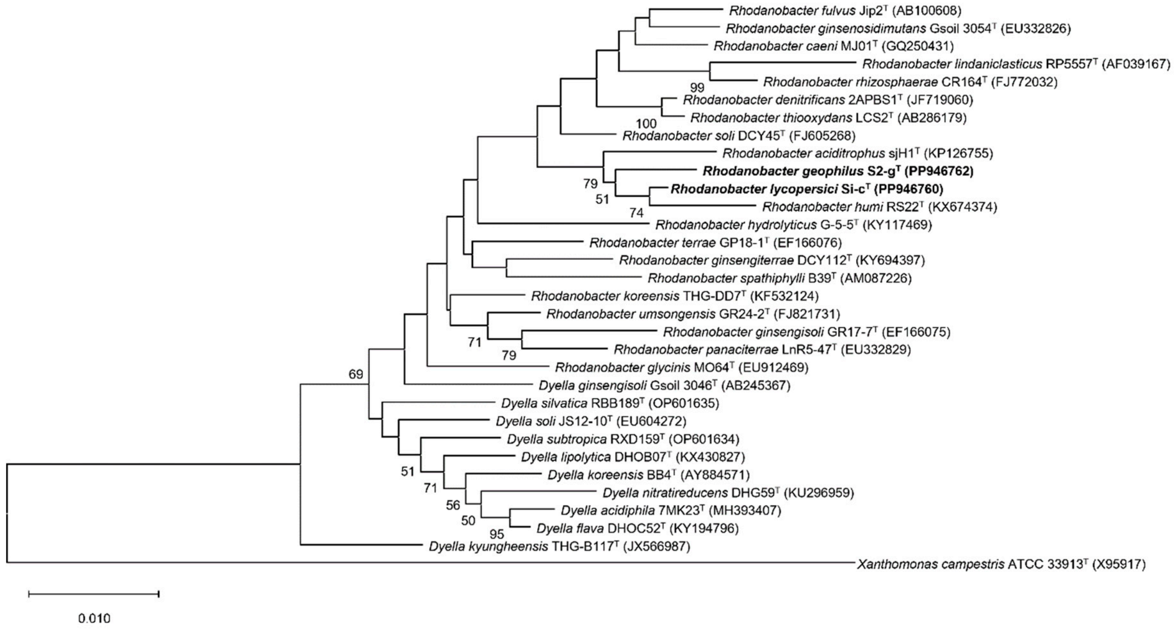

(b) MP tree

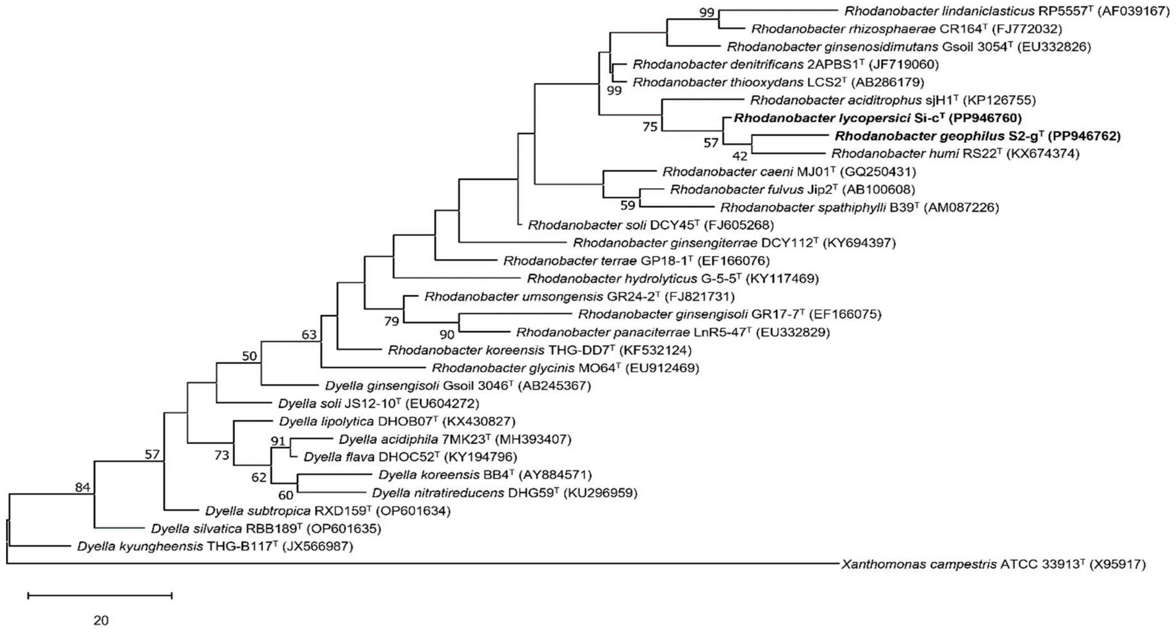

Figure S2. AAI tree generated using EzAAI illustrating the phylogenetic positions of the strains Si-c^{T}, S2-g^{T}, and their closely related species. Bar represents 5 substitutions per nucleotide position. *Xanthomonas campestris* ATCC 33913^{T} was selected as an outgroup.

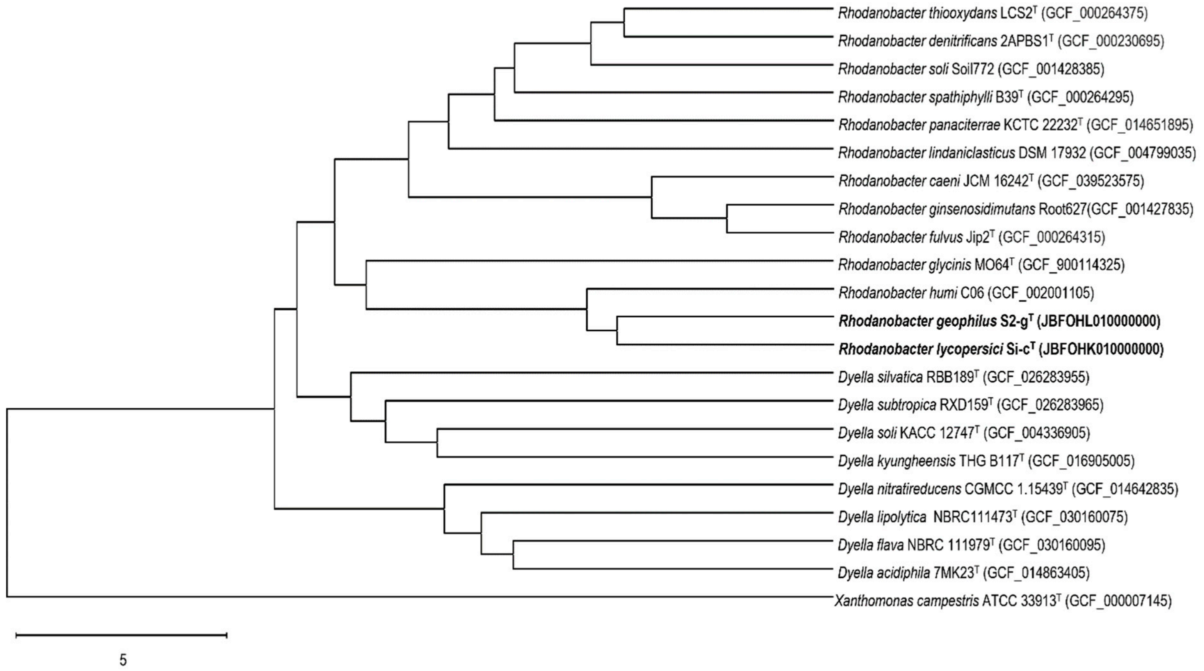

Figure S3. Graphical circular genomic map generated using the Proksee online server, displaying the genome views for strains (a) Si-c^T and (b) S2-g^T. From the center to the periphery: rings 1 and 2 represent G+C skew; ring 3 represents G+C content; rings 4 and 6 represent CDSs; ring 5 represents the contigs; ring 6 represents CRISPRs; rings 7 and 8 represent mobile genetic elements; ring 9 represents comprehensive antibiotic resistance.

(a) Genomic map of strain Si-c^T

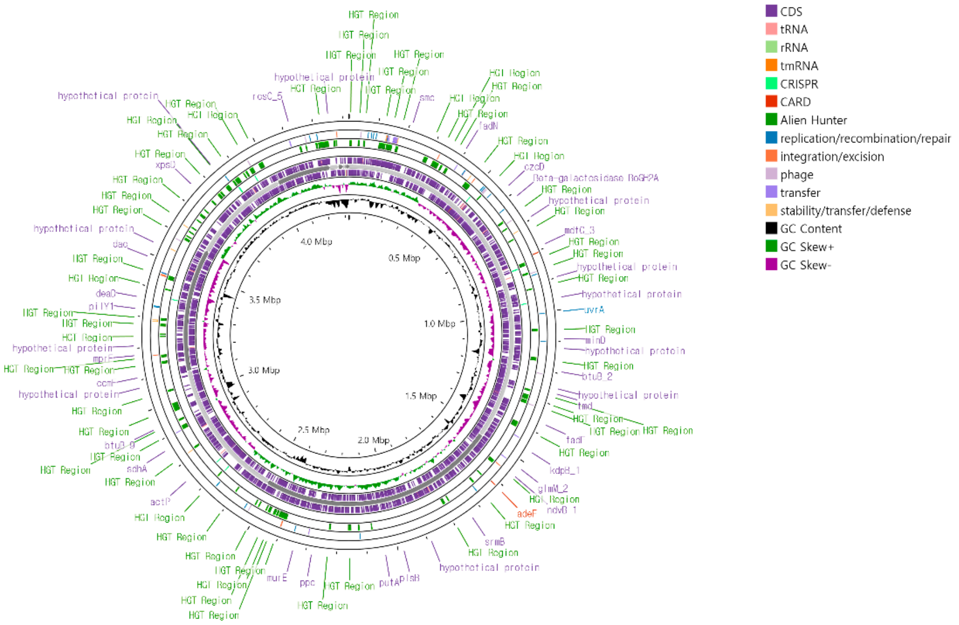

(b) Genomic map of strain S2-g^T

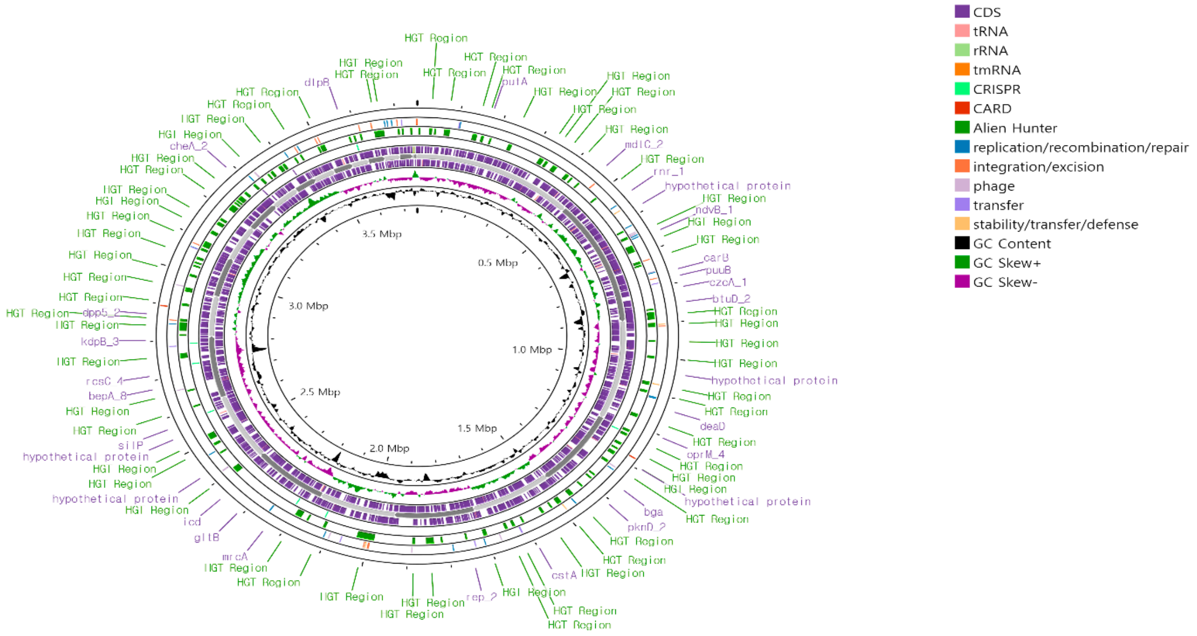

Figure S4. Venn diagrams built using the OrthoVenn3 online server, exhibiting the number of shared and unique orthologous gene clusters of strains Si-c^{T}, S2-g^{T}, and other closely related species. 1, Si-c^{T}; 2, S2-g^{T}; 3, *R. humi* C06; 4, *R. denitrificans* 2APBS1^{T}; 5, *R. thiooxydans* LCS2^{T}.

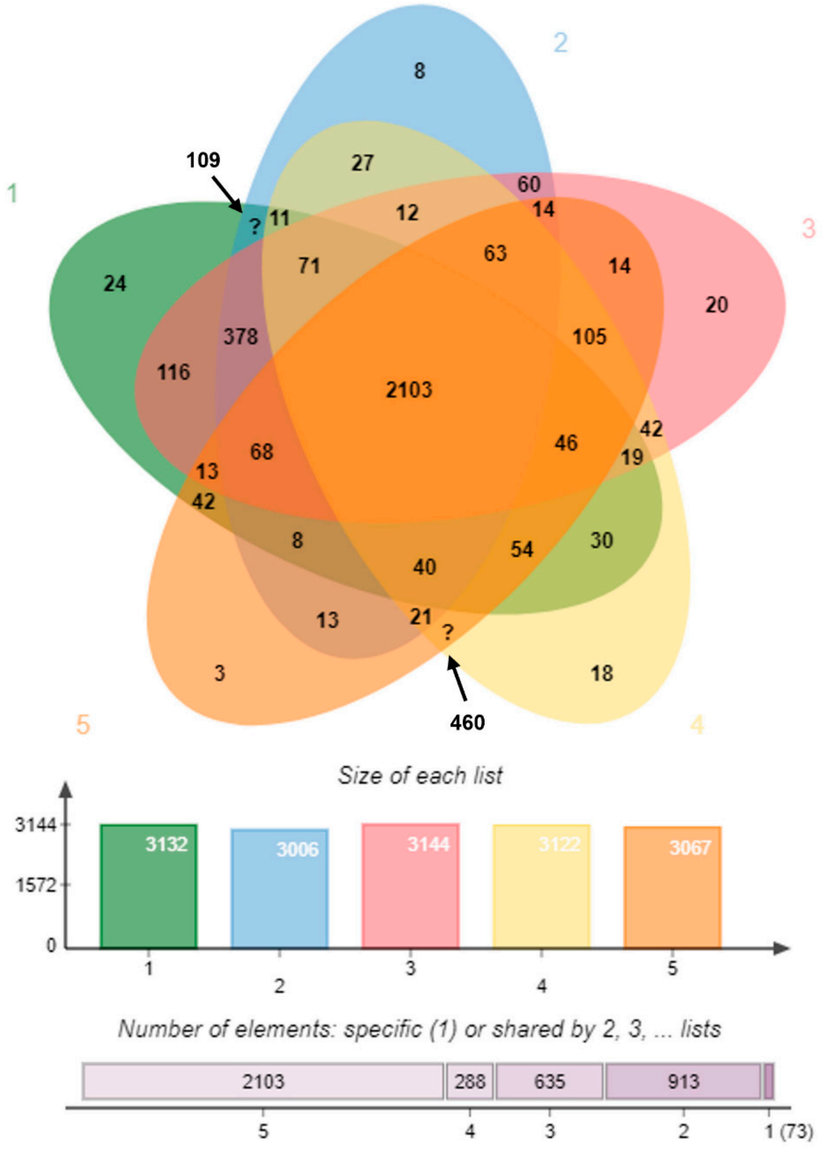

Figure S5. Cell morphology under the transmission electron microscope for (a) Si-c^{T} and (b) S2-g^{T}.

(a) Strain Si-c^{T}

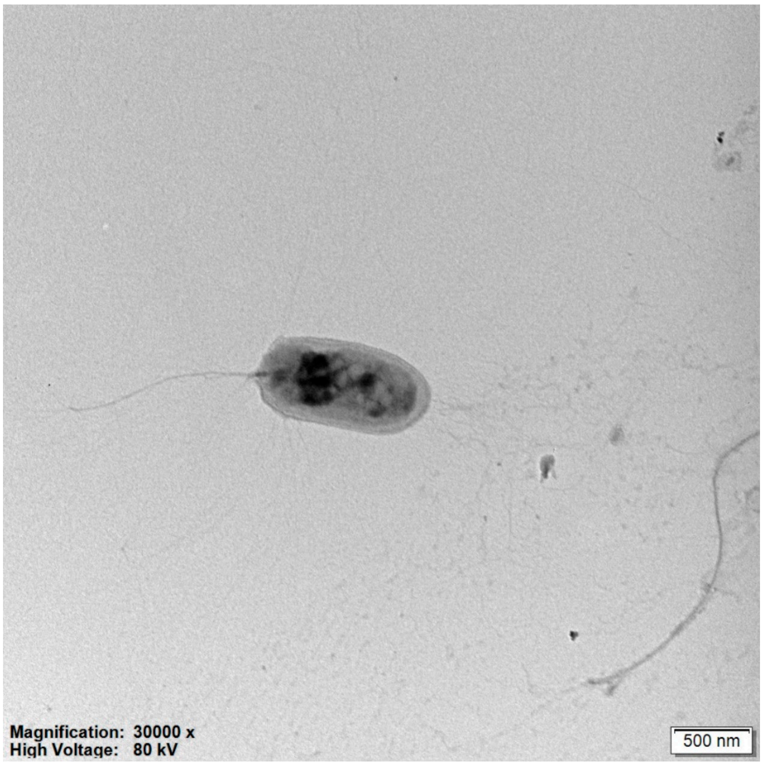

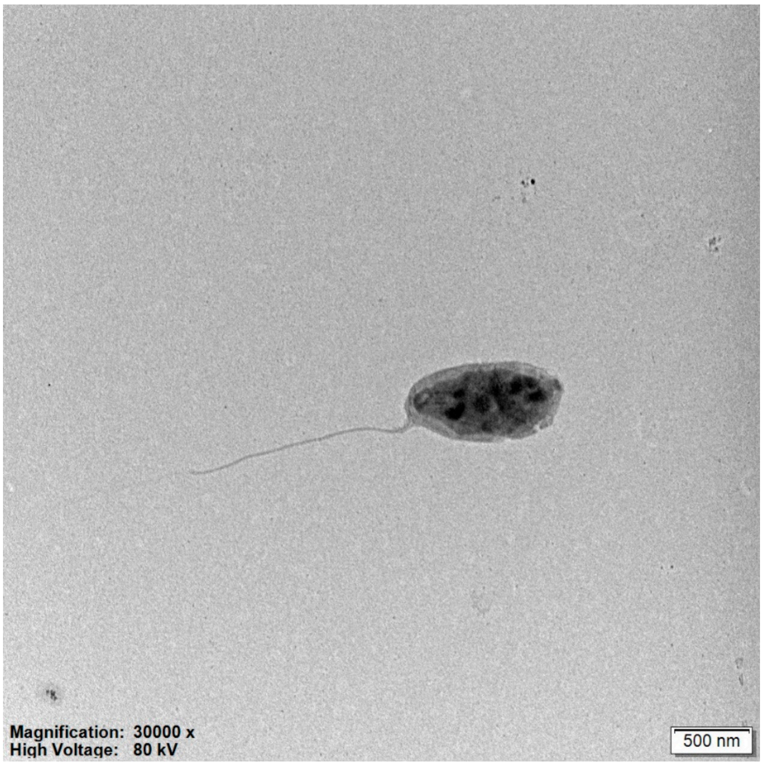

(B) Strain S2-g^T

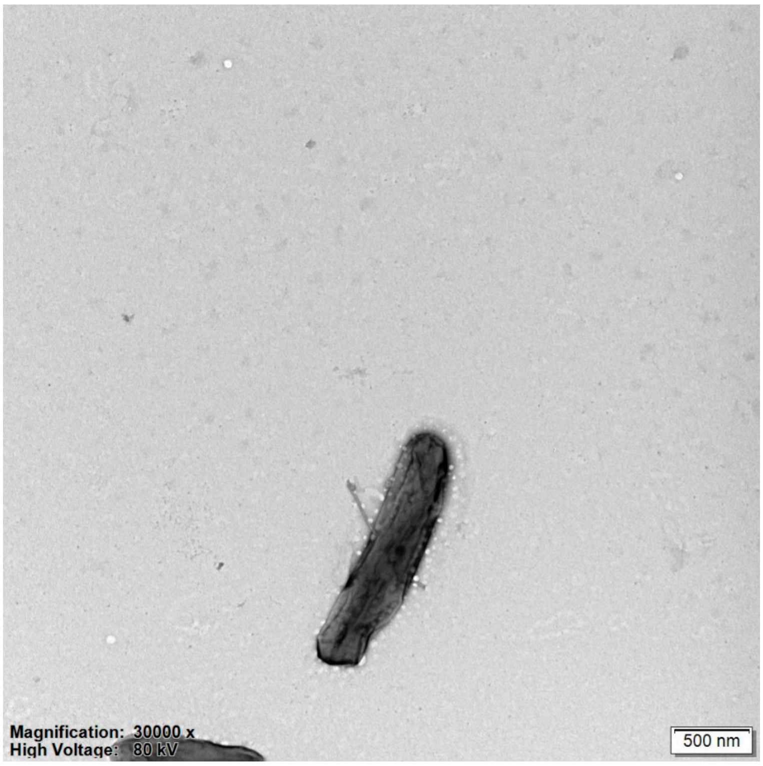

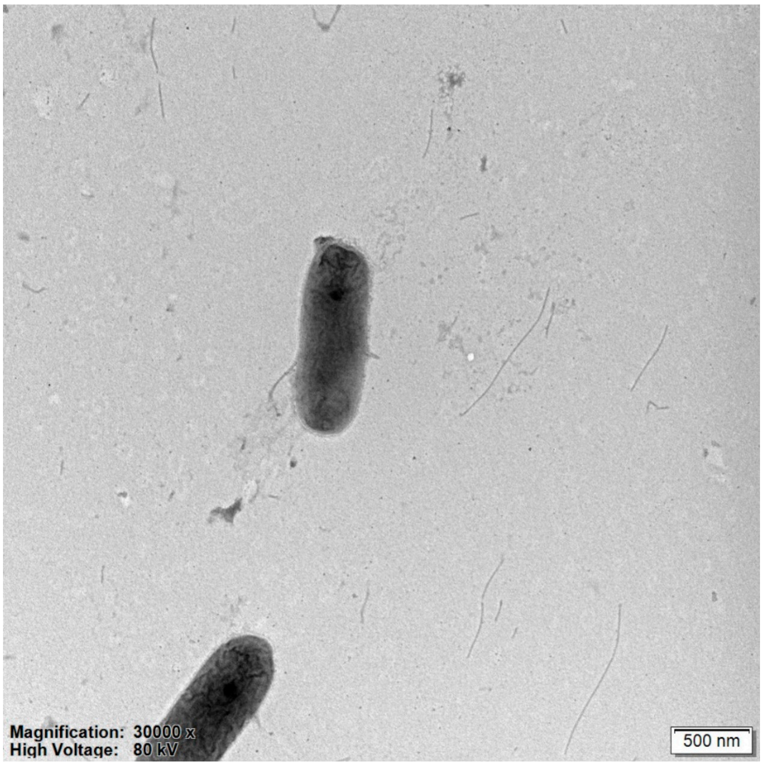

Figure S6. Polar lipid profiles of strains (a) Si-c^T and (b) S2-g^T. PE, Phosphatidylethanolamine; DPG, diphosphatidylglycerol; PME, phosphatidyl-N-methylethanolamine; PG, phosphatidylglycerol; APGL, aminophosphoglycolipid; APL, aminophospholipid; PGL, phosphoglycolipid; GL, glycolipid; PL, phospholipid.

(a) Strain Si-c^T

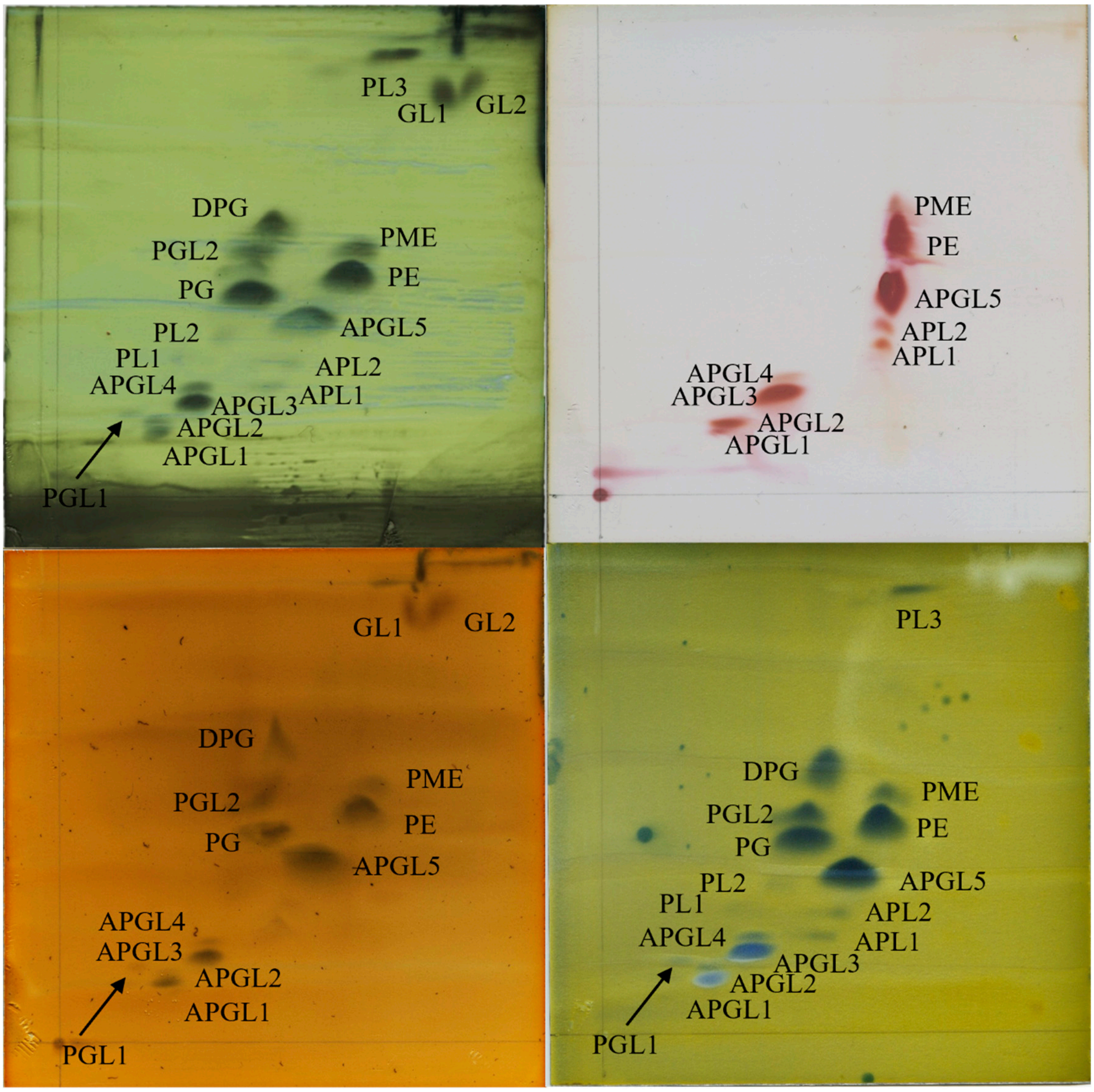

(b) Strain S2-g^{T}

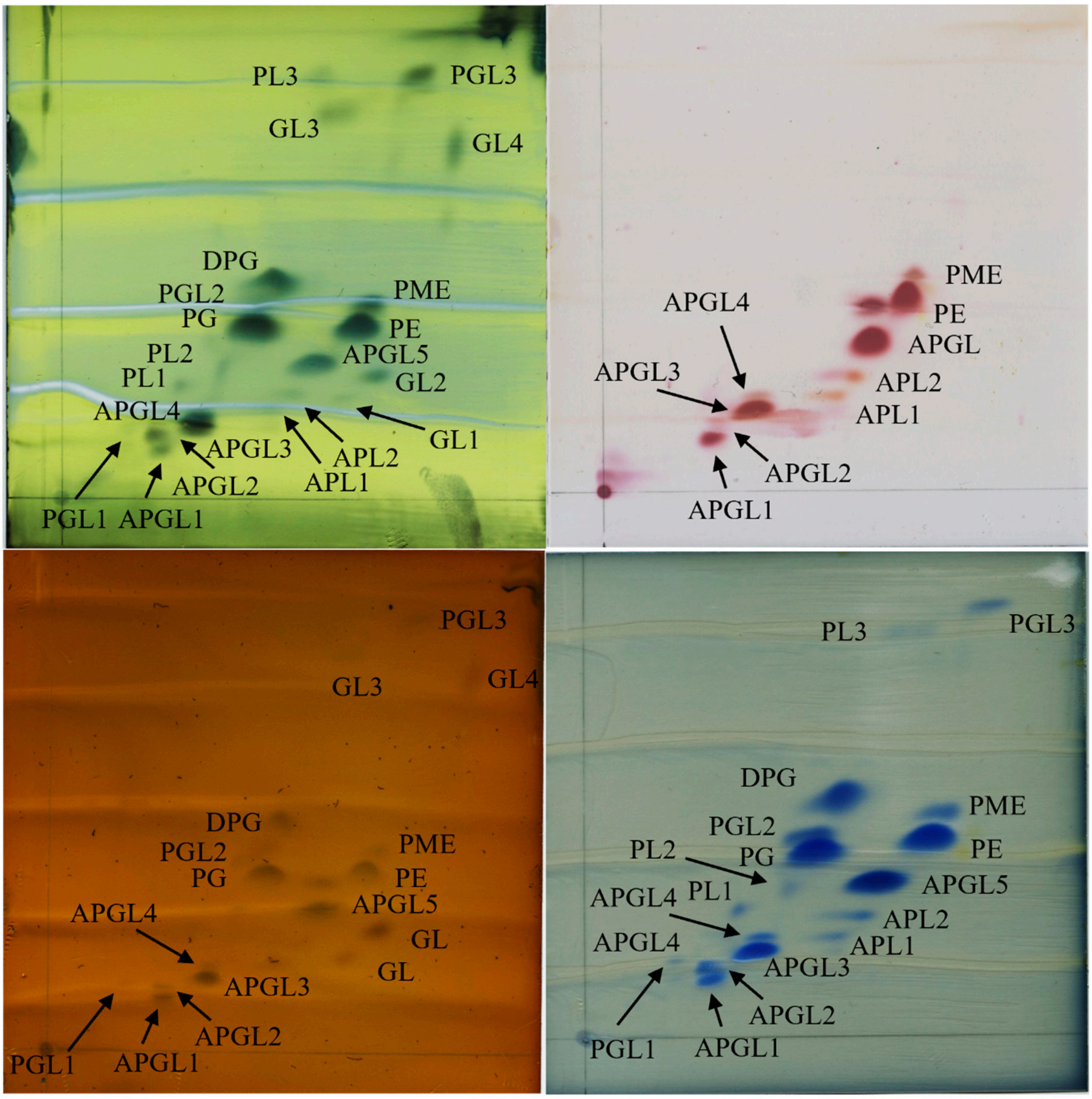

Table S1. Overview of flagella-related genes in the genomes of strains Si-c^T, S2-g^T, and closely related species within the genus *Rhodanobacter*. 1, Si-c^T; 2, S2-g^T; 3, *R. humi* C06; 4, *R. denitrificans* 2APBS1^T; 5, *R. thiooxydans* LCS2^T.

Gene	Protein name	Protein Product				
		1	2	3	4	5
flgA	flagellar basal body P-ring formation chaperone	WP_367854894.1	WP_367845737.1	WP_077480838.1	WP_015448522.1	WP_008434139.1
flgB	flagellar basal body rod protein	WP_367854891.1	WP_367845740.1	WP_077480832.1	WP_015448519.1	WP_008434144.1
flgC	flagellar basal body rod protein	WP_367854890.1	WP_367845741.1	WP_077480830.1	WP_015448518.1	WP_008434145.1
flgE	flagellar hook protein	WP_367854888.1	WP_367845743.1	WP_077480826.1	WP_015448516.1	WP_008434147.1
flgF	flagellar basal-body rod protein	WP_367854887.1	WP_367845744.1	WP_077480824.1	WP_015448515.1	WP_081491494.1
flgG	flagellar basal-body rod protein	WP_367854886.1	WP_367845745.1	WP_077480822.1	WP_015448514.1	WP_008434149.1
flgH	flagellar basal body L-ring protein	WP_367854913.1	WP_367845746.1	WP_254426658.1	WP_015448513.1	WP_008434151.1
flgJ	flagellar assembly peptidoglycan hydrolase	WP_367854884.1	WP_367845748.1	WP_077480820.1	WP_015448511.1	WP_008434155.1
flgK	flagellar hook-associated protein	WP_367854883.1	WP_367845749.1	WP_077480818.1	WP_015448510.1	WP_008434157.1
flgL	flagellar hook-associated protein	WP_367854882.1	WP_367845750.1	WP_254426657.1	WP_015448509.1	WP_008434159.1
flgM	flagellar biosynthesis anti-sigma factor	WP_367854895.1	WP_367845736.1	WP_077480840.1	WP_007511401.1	WP_008434137.1
flhA	flagellar biosynthesis protein	WP_367854858.1	WP_367845776.1	WP_077483977.1	WP_007506959.1	WP_008439154.1
flhB	flagellar biosynthesis protein	WP_367854859.1	WP_367845775.1	WP_077483976.1	WP_007506960.1	WP_008439155.1
flhF	flagellar biosynthesis protein	WP_367854857.1	WP_367845777.1	WP_077483978.1	WP_015448496.1	WP_008439153.1
fliD	flagellar filament capping protein	WP_367854877.1	WP_367845755.1	WP_077483958.1	WP_015448507.1	WP_008434162.1
fliE	flagellar hook-basal body complex protein	WP_367854872.1	WP_367845761.1	WP_077483963.1	WP_015448504.1	WP_008434170.1
fliF	flagellar basal-body MS-ring/collar protein	WP_367854871.1	WP_367845762.1	WP_077483964.1	WP_007511355.1	WP_008434172.1
fliG	flagellar motor switch protein	WP_367854870.1	WP_367845763.1	WP_077483965.1	WP_015448503.1	WP_008434174.1
fliI	flagellar protein export ATPase	WP_367854868.1	WP_367845765.1	WP_077483967.1	WP_015448502.1	WP_008434177.1
fliJ	flagellar export protein	WP_367854867.1	WP_367845766.1	WP_077483968.1	WP_015448501.1	WP_008434179.1
fliL	flagellar basal body-associated protein	WP_367854865.1	WP_367845768.1	–	–	–
fliM	flagellar motor switch protein	WP_367854864.1	WP_367845769.1	WP_077483970.1	WP_007515028.1	WP_008439169.1
fliN	flagellar motor switch protein	WP_367854863.1	WP_367845770.1	WP_077483971.1	WP_007515026.1	WP_008439167.1
fliO	flagellar biosynthetic protein	WP_367854862.1	WP_367845771.1	WP_077483972.1	WP_015448498.1	WP_235605904.1
fliP	flagellar type III secretion system pore protein	WP_367854912.1	WP_367845772.1	WP_077483973.1	WP_015448497.1	WP_008439164.1
fliQ	flagellar biosynthesis protein	WP_367854861.1	WP_367845773.1	WP_077483974.1	WP_007506969.1	WP_008439158.1
fliR	flagellar biosynthetic protein	WP_367854860.1	WP_367845774.1	WP_077483975.1	WP_007506961.1	WP_008439157.1
fliS	flagellar export chaperone	WP_367854876.1	WP_367845756.1	WP_077483959.1	WP_007511364.1	WP_008434164.1
fliT	flagellar protein	–	–	–	WP_236126959.1	–
motD	flagellar motor protein	WP_367854850.1	WP_367845784.1	WP_077483984.1	WP_015448492.1	WP_008439000.1

Table S2. Genes related to pH tolerance of strains (a) Si-c^T and (b) S2-g^T identified with NCBI annotation systems.

(a) Genes related to pH tolerance of strain Si-c^T

Gene	Protein Name	Accession	Start	Stop	Strand	Length (aa)
atpA	F0F1 ATP synthase subunit alpha	JBFOHK010000002	312582	314321	+	516
atpB	F0F1 ATP synthase subunit A	JBFOHK010000002	310148	310969	+	273
atpD	F0F1 ATP synthase subunit beta	JBFOHK010000002	315271	316704	+	477
atpE	F0F1 ATP synthase subunit C	JBFOHK010000002	311032	311319	+	95
atpG	F0F1 ATP synthase subunit gamma	JBFOHK010000002	314195	315112	+	305
phoB	phosphate regulon transcriptional regulator PhoB	JBFOHK010000004	421015	421704	+	229
phoR	phosphate regulon sensor histidine kinase PhoR	JBFOHK010000004	422042	423091	+	349
phoU	phosphate signaling complex protein PhoU	JBFOHK010000001	638194	638925	+	243
cyoA	ubiquinol oxidase subunit II	JBFOHK010000001	549546	550466	-	306
cyoA	ubiquinol oxidase subunit II	JBFOHK010000001	1431512	1432495	+	327
cyoB	cytochrome o ubiquinol oxidase subunit I	JBFOHK010000001	547527	549542	-	671
cyoB	cytochrome o ubiquinol oxidase subunit I	JBFOHK010000001	1432518	1434548	+	676
cyoC	cytochrome o ubiquinol oxidase subunit III	JBFOHK010000001	546910	547524	-	204
cyoC	cytochrome o ubiquinol oxidase subunit III	JBFOHK010000001	1434545	1435162	+	205
cyoD	cytochrome o ubiquinol oxidase subunit IV	JBFOHK010000001	546563	546910	-	115
cyoD	cytochrome o ubiquinol oxidase subunit IV	JBFOHK010000001	1435159	1435530	+	123
cyoE	heme o synthase	JBFOHK010000002	540029	540934	+	301
ccoN	cytochrome-c oxidase, cbb3-type subunit I	JBFOHK010000001	1457164	1458630	+	488
ccoO	cytochrome-c oxidase, cbb3-type subunit II	JBFOHK010000001	1458641	1459294	+	217
ccoP	cytochrome-c oxidase, cbb3-type subunit III	JBFOHK010000001	1459470	1460381	+	303
ccoS	cbb3-type cytochrome oxidase assembly protein CcoS	JBFOHK010000001	1462796	1462984	+	62
dnaK	molecular chaperone DnaK	JBFOHK010000001	434253	436172	-	639
clpA	ATP-dependent Clp protease ATP-binding subunit ClpA	JBFOHK010000001	42355	44628	+	757
clpB	ATP-dependent chaperone ClpB	JBFOHK010000003	7552	10134	+	860
clpP	ATP-dependent Clp endopeptidase proteolytic subunit ClpP	JBFOHK010000001	528195	528818	-	207
clpS	ATP-dependent Clp protease adapter ClpS	JBFOHK010000001	41931	42257	+	108
clpX	ATP-dependent Clp protease ATP-binding subunit ClpX	JBFOHK010000001	526723	528015	-	430
pstA	phosphate ABC transporter permease PstA	JBFOHK010000001	636381	637250	+	289
pstB	phosphate ABC transporter ATP-binding protein PstB	JBFOHK010000001	637243	638058	+	271
pstC	phosphate ABC transporter permease subunit PstC	JBFOHK010000001	635410	636381	+	323
pstS	phosphate ABC transporter substrate-binding protein PstS	JBFOHK010000001	634202	635218	+	338
rpoE	RNA polymerase sigma factor RpoE	JBFOHK010000001	500046	500660	-	204

(b) Genes related to pH tolerance of strain S2-g^{T}

Gene	Protein Name	Accession	Start	Stop	Strand	Length (aa)
atpA	F0F1 ATP synthase subunit alpha	JBFOHL010000001	366753	368303	-	516
atpB	F0F1 ATP synthase subunit A	JBFOHL010000001	369957	370772	-	271
atpD	F0F1 ATP synthase subunit beta	JBFOHL010000001	364256	365692	-	478
atpE	F0F1 ATP synthase subunit C	JBFOHL010000001	369617	369895	-	92
atpG	F0F1 ATP synthase subunit gamma	JBFOHL010000001	365773	366690	-	305
phoB	phosphate regulon transcriptional regulator PhoB	JBFOHL010000012	65413	66102	-	229
phoR	phosphate regulon sensor histidine kinase PhoR	JBFOHL010000012	64073	65371	-	432
phoU	phosphate signaling complex protein PhoU	JBFOHL010000012	71639	72370	-	243
cyoA	ubiquinol oxidase subunit II	JBFOHL010000002	147941	148909	+	322
cyoA	ubiquinol oxidase subunit II	JBFOHL010000010	165231	166238	+	335
cyoB	cytochrome o ubiquinol oxidase subunit I	JBFOHL010000002	148913	150928	+	671
cyoB	cytochrome o ubiquinol oxidase subunit I	JBFOHL010000010	166303	168291	+	662
cyoC	cytochrome o ubiquinol oxidase subunit III	JBFOHL010000002	150933	151547	+	204
cyoC	cytochrome o ubiquinol oxidase subunit III	JBFOHL010000010	168288	168914	+	208
cyoD	cytochrome o ubiquinol oxidase subunit IV	JBFOHL010000002	151547	151894	+	115
cyoD	cytochrome o ubiquinol oxidase subunit IV	JBFOHL010000010	168911	169288	+	125
cyoE	heme o synthase	JBFOHL010000001	161798	162703	-	301
ccoN	cytochrome-c oxidase, cbb3-type subunit I	JBFOHL010000010	188750	190213	+	487
ccoO	cytochrome-c oxidase, cbb3-type subunit II	JBFOHL010000010	190434	191084	+	216
ccoP	cytochrome-c oxidase, cbb3-type subunit III	JBFOHL010000010	191260	192171	+	303
ccoS	cbb3-type cytochrome oxidase assembly protein CcoS	JBFOHL010000010	194592	194780	+	62
dnaK	molecular chaperone DnaK	JBFOHL010000002	282894	284813	+	639
clpA	ATP-dependent Clp protease ATP-binding subunit ClpA	JBFOHL010000009	24199	26472	-	757
clpB	ATP-dependent chaperone ClpB	JBFOHL010000007	151150	153732	+	860
clpP	ATP-dependent Clp endopeptidase proteolytic subunit ClpP	JBFOHL010000002	169388	170011	+	207
clpS	ATP-dependent Clp protease adapter ClpS	JBFOHL010000009	26537	26863	-	108
clpX	ATP-dependent Clp protease ATP-binding subunit ClpX	JBFOHL010000002	170184	171476	+	430
pstA	phosphate ABC transporter permease PstA	JBFOHL010000002	73333	74202	-	289
pstB	phosphate ABC transporter ATP-binding protein PstB	JBFOHL010000002	72513	73340	-	275
pstC	phosphate ABC transporter permease subunit PstC	JBFOHL010000002	74202	75173	-	323
pstS	phosphate ABC transporter substrate-binding protein PstS	JBFOHL010000002	75395	76429	-	344
rpoE	RNA polymerase sigma factor RpoE	JBFOHL010000002	227156	227770	+	204

Table S3. Comparison of subsystem category distribution and feature counts for strains Si-c^T, S2-g^T, and other closely related taxa using RAST analysis. 1, Si-c^T; 2, S2-g^T; 3, *R. humi* C06; 4, *R. denitrificans* 2APBS1^T; 5, *R. thiooxydans* LCS2^T.

	1	2	3	4	5
Amino Acids and Derivatives	230	234	243	227	238
Carbohydrates	164	144	183	160	177
Cell Wall and Capsule	26	25	26	29	26
Cell Division and Cell Cycle	0	0	0	0	0
Cofactors, Vitamins, Prosthetic Groups, Pigments	158	126	144	139	135
DNA Metabolism	56	65	61	84	81
Dormancy and Sporulation	1	1	1	1	1
Fatty Acids, Lipids, and Isoprenoids	52	45	51	87	96
Iron acquisition and metabolism	1	0	1	0	0
Membrane Transport	64	65	91	106	105
Metabolism of Aromatic Compounds	19	12	12	19	19
Miscellaneous	18	14	21	14	16
Motility and Chemotaxis	15	20	54	20	20
Nitrogen Metabolism	23	9	25	40	43
Nucleosides and Nucleotides	60	54	56	61	66
Phages, Prophages, Transposable elements, Plasmids	1	1	1	2	3
Phosphorus Metabolism	24	21	22	23	23
Photosynthesis	0	0	0	0	0
Potassium metabolism	14	13	11	7	7
Protein Metabolism	182	178	193	192	174
Regulation and Cell signaling	11	6	8	12	12
Respiration	89	86	88	81	83
RNA Metabolism	40	37	40	41	42
Secondary Metabolism	4	4	4	4	4
Stress Response	63	67	78	79	60
Sulfur Metabolism	16	19	18	16	16
Virulence, Disease and Defense	26	24	28	64	56

Table S4. Overview of identified BGCs in the (a) Si-c^T and (b) S2-g^T genome predicted by antiSMASH.

(a) BGC of strain Si-c^T

Cluster serial no.	Region	Cluster type	Size (nt)	Details of the most similar known cluster			
				Product	Biosynthetic class	MIBiG accession	Similarity (%)
1	1.1	arylpolyene	48488	APE Vf	Other	BGC0000837	30
2	1.2	terpene	20855				
3	2.1	arylpolyene	41197	lysobactin	NRP		30
4	2.2	RiPP-like	10834				
5	5.1	NRPS, T1PKS	58930	O-antigen	Saccharide	BGC0000781	14
6	8.1	lassopeptide	19432				

(B) BGC of strain S2-g^T

Cluster serial no.	Region	Cluster type	Size (nt)	Details of the most similar known cluster			
				Product	Biosynthetic class	MIBiG accession	Similarity (%)
1	1.1	arylpolyene	41197	lysobactin	NRP	BGC0000385	30
2	4.1	arylpolyene	48674	APE Ec	Other	BGC0000836	36
3	4.2	terpene	20852				
4	8.1	T1PKS, NRPS-like, NRPS	28854	O-antigen	Saccharide	BGC0000781	14
5	13.1	NRPS, NRPS-like	24539	rhizomide A/B/C	NRP	BGC0001758	100
6	20.1	lassopeptide	21635	xanthomonin I/II	RiPP	BGC0000580	50
7	25.1	NRPS	2244				

Table S5. Fatty acid compositions (% of the total) of strains Si-c^T, S2-g^T, and closely related taxa. Fatty acids constituting less than 1.0% in all strains were excluded, so the total percentages may not sum to 100%. Values greater than 10 were highlighted in bold. Strains: 1, Si-c^T; 2, S2-g^T; 3, *R. humi* RS22^T; 4, *R. denitrificans* 2APBS1^T; and 5, *R. thiooxydans* LCS2^T. –, not detected or <1.0%.

Fatty acid	1	2	3	4	5
**Saturated:**					
$C_{16:0}$	5.2	7.4	5.3	1.9	1.9
$C_{18:0}$	1.0	1.3	–	–	–
**Branched saturated:**					
iso-$C_{11:0}$	2.7	2.7	4.8	3.1	4.1
iso-$C_{15:0}$	**17.3**	**18.6**	**20.7**	**21.4**	**31.8**
iso-$C_{16:0}$	**13.6**	9.6	8.4	**11.1**	4.6
iso-$C_{17:0}$	**15.8**	**10.6**	**13.7**	**14.4**	**10.6**
iso-$C_{18:0}$	1.4	–	–	–	–
anteiso-$C_{15:0}$	4.6	5.6	6.0	5.2	5.4
anteiso-$C_{17:0}$	2.0	2.5	–	–	–
**Hydroxy:**					
iso-$C_{11:0}$ 3-OH	5.3	5.2	4.3	3.6	3.3
iso-$C_{13:0}$ 3-OH	2.9	2.9	2.4	1.7	2.7
**Summed features***					
3	2.0	3.2	3.8	4.1	5.7
8	–	2.5	–	–	–
9	**19.4**	**21.3**	**25.7**	**24.6**	**24.3**

*Summed features represent two or more fatty acids that are not separated using the selected chromatographic conditions. Summed feature 3 comprises $C_{16:1}$ *ω7c* and/or $C_{16:1}$ *ω6c*. Summed feature 8 comprises $C_{18:1}$ *ω7c* and/or $C_{18:1}$ *ω6c*. Summed feature 9 comprises iso-$C_{17:1}$ *ω9c* and/or $C_{16:0}$ *ω6c* 10-methyl.
